# The additive from co-fermented edible plants and probiotics improved calves’ growth performance and health by regulating antioxidant and gastrointestinal-microbiota

**DOI:** 10.5713/ab.250112

**Published:** 2025-11-14

**Authors:** Yi-Ou Xu, Qing-Hua Wu, Xiang-Long Zhang, Xiu-Jie Yin, Yong-Gen Zhang, Yang Li, Xiu-Jing Dou

**Affiliations:** 1 College of Animal Science and Technology, Northeast Agricultural University, Harbin, China

**Keywords:** Dairy Calf, Disease Resistance, Edible Plants, Gastrointestinal Microbiota, Growth Performance, Probiotics

## Abstract

**Objective:**

The study aimed to assess effects of supplemented co-fermented edible plants and probiotics (AEPP) on growth performance, disease resistance, plasma and rumen metabolites, and bacterial communities in the rumen and feces of pre-weaned calves.

**Methods:**

Twenty female Holstein calves (7±0.50 d, 41.65±6.20 kg) were randomly assigned to one of two treatments: the control group or the treatment group (30 g/head/day AEPP supplementation). Growth performance, blood, and fecal samples were measured on regular basis. On day 30 of the trial, rumen fluid and fecal samples were collected for multi-omics analysis.

**Results:**

Dietary supplementation with AEPP enhanced calf growth and improved disease resistance, as evidenced by a reduced incidence of respiratory disease and diarrhea and a decreased frequency of antibiotic therapy (p<0.05). The treatment group exhibited enrichment of rumen microorganisms *Prevotella*, *Ruminococcus*, and *Xylanibacter* (linear discriminant analysis>2, p<0.05), along with increased activity in beneficial metabolites such as indoleacetic acid, which activated starch and sucrose metabolism and tryptophan metabolism pathway. This intervetion significantly improved average daily gain, feed efficiency, immunoglobulin G, total superoxide dismutase, and glutathione peroxidase activities, as well as significantly reduced levels of tumor necrosis factor-alpha and interleukin-6 (p<0.05), promoting calf growth and health. The elevated abundance of fecal microorganisms, *Subdoligranulum* and *Bifidobacterium*, in the treatment group altered fecal pH, short-chain fatty acids, and butyrate proportions (p<0.05).

**Conclusion:**

Feeding AEPP improved growth performance, disease resistance, and antioxidant function. It altered the bacterial communities and metabolic profiles in the rumen and feces of preweaning dairy calves, providing a data reference for the use of AEPP in young ruminant production.

## INTRODUCTION

Newborn calves serve as a crucial reserve in dairy farms. Their growth rate and basic protein deposition at this stage are key to milk performance in the future [1]. It is known that the antibodies from passive transfer of calves reach the lowest value at 14 to 21 days, while the establishment of an active immune system is not yet complete, and the rising risk of pathogen invasion, resulting in a high incidence of diarrhea, pneumonia, and other diseases and an increase in calf mortality, posing the risk of great economic loss [2]. Therefore, the health of calves within 30 days of birth is critical for survival.

In previous practical production, antibiotics have played an important role in helping livestock recover. However, given the prolonged administration of antibiotics, resulting in the onset of pathogenic microorganism resistance, besides the presence of residual antibiotics in animal-derived products, there has been a persistent scientific focus on exploring antibiotic alternatives. At present, the use of antibiotic substitutes in production includes edible plants, extracts, probiotics, etc. Edible plants are defined as the products obtained by drying, crude extraction, or crushing of the plant or specific parts of the plant, which are relatively difficult to develop drug resistance, and have antibacterial, antioxidant, growth promotion, and other beneficial effects [3]. Furthermore, direct-fed microbial (DFM) is a dietary supplement that regulates the intestinal microbial environment, reduces the invasion of harmful substances, and promotes body growth. *Terminalia chebula (T. chebula)* and *Cirsium setosum* (Willd.) MB (*C. setosum* [Willd.] MB) are Chinese feed categorized as edible plants that can be fed directly to livestock in *the Catalogue of Feed Materials of China*. *T. chebula* and *C. setosum* (Willd.) MB have been demonstrated to possess beneficial antibacterial properties, resist inflammatory responses, and reduce diarrhea and respiratory illnesses. These effects may be related to their rich contents of flavonoids, triterpenoids, and other bioactive components [4,5], which could be the main active ingredients. Edible plants, abundant in flavonoids and terpenoids, have been proven to strengthen young ruminants’ immune system and antioxidant capacity, thereby reducing morbidity rates, enhancing overall health, and improving growth outcomes [6,7]. However, plants may hinder the release of intracellular nutrients due to their unique cell wall structure, thereby affecting the digestion and absorption of active ingredients by animals. In addition, these untreated edible plants may affect the willingness and feed intake of livestock due to their bitterness. Research indicates that *Bacillus subtilis (B. subtilis)* as DFM can synthesize hemicellulase during its growth and metabolism, facilitating the breakdown of hemicellulose [8]. This process facilitates the spillover of bioactive compounds blocked by the cell wall, thereby enhancing the efficacy of the biological functions in edible plants. Some studies have discovered it could support the colonization and growth of intestinal microbiota, mitigate the invasion of harmful pathogens, enhance intestinal health, lower the likelihood of diarrhea, and promote overall calf health with the supplementation of *Lactobacillus plantarum* (*L. plantarum*) and *Saccharomyces cerevisiae* (*S. cerevisiae*) [7,9]. Therefore, utilizing probiotics fermentation processes with edible plants as feed additives directly for livestock consumption can significantly enhance palatability and stimulate appetite for feed. It addresses the limitations of the efficacy of edible plants, thereby demonstrating synergistic potential in enhancing overall feed intake and controlling calf diseases. Currently, limited studies have jointly evaluated variables associated with calf health through the combined effects of edible plants and probiotics on fecal fermentation parameters and microbial communities. We hypothesized that the additive from co-fermented edible plants and probiotics (AEPP) may optimize calf growth performance and resilience to disease by bolstering intestinal health and immunity. Consequently, the objective of this study is to evaluate the impacts of AEPP on growth performance, morbidity rates, volatile fatty acids (VFA), plasma and rumen metabolites, and bacterial communities in the rumen and feces, aiming to furnish a theoretical foundation and data validation for integrating the new feed additive into calf production.

## MATERIALS AND METHODS

### Processing of the additive from co-fermented edible plants and probiotics

AEPP consisted of *T. chebula* and *C. setosum* (Willd.) MB, *S. cerevisiae*, *B. subtilis*, and *L. plantarum*. The development of the strains and the production process of AEPP were carried out by Harbin PROSYN Unite Microbial Feed. *S. cerevisiae* (GDMCC NO. 2.167) and *B. subtilis* (GDMCC NO. 1.372) were from the Guangdong Microbial Culture Collection Center, and *L. plantarum* (*Lp*90) was isolated from pickled radish and had been previously kept at Jiangsu Wecare Biotechnology. *T. chebula* and *C. setosum* (Willd.) MB were purchased from Nanjing Tongrentang, and identified by Prof. Yin xiujie, Department of Grassland Science, Northeast Agricultural University, as the whole herb of *T. chebula Retz*., family Combretaceae and *C. setosum* (Willd.) MB of *Cirsium japonicum*, family Asteraceae.

The 0.1 mL sterile water was sucked with sterile pipettes into three types of lyophilized bacterial powder in the ampoule. They were shaken gently to dissolve the lyophilized bacteria in suspension. *S. cerevisiae*, *L. plantarum*, and *B. subtilis* were incubated in YPD medium, Bacillus medium, and MRS medium (Qingdao Hope Bio-Technology) at 30°C, 37°C, and 37°C, respectively, for 24 h. The strain suspensions were subjected to 10-fold serial dilutions, and each dilution was spread evenly onto their respective solid media. Subsequently, the plates were incubated under the same conditions for 24 h to determine the colony-forming units. The results showed *S. cerevisiae* had 1.0×10^9^ cfu/mL, *L. plantarum* had 2.0×10^9^ cfu/mL, and *B. subtilis* had 2.5×10^9^ cfu/mL.

All strains were mixed to obtain the compound probiotic liquid extract, and it was sprayed together with distilled water into a temperature-controlled fermenter with constant stirring (Nanjing Runzhe Bioengineering Equipment) containing fermented substrate and glucose (1.0%). The substrate consisted of edible plants (10% *T. chebula* and 15% *C. setosum* (Willd.) MB), 50% corn, and 25% soybean meal. The fermenter began with a temperature range of 35°C to 42°C, and the moisture content throughout the fermentation process was consistently maintained at approximately 37%. Then, the fermenter was sealed tightly, the temperature was adjusted to 65°C, and fermentation was carried out for 60 h to obtain AEPP. After fermentation was completed, AEPP was ground, sieved through a 1-mm mesh, and continuously stirred until it was mixed evenly. The contents of bioactive ingredients in AEPP were assayed at Sinotest Certification Group. The composition of total flavonoids and total triterpenes was evaluated by using the NaNO_2_–Al(NO_3_)_3_ colorimetric methods and the spectrophotometric method with ursolic acid as a standard substance, respectively [10,11]. The main contents of plant secondary metabolites and the strain number of live probiotics in AEPP are shown in Table 1. Some loss of probiotics occurred during fermentation.

### Animals, experimental design, and diets

The experiment was carried out at Niuniu Husbandry from October to December 2022. The newborn calves on the dairy farm were all offspring of multiparous cows of three parities. They were immediately transported to individual calf hutches (2,200×1,450×1,490 mm) bedded with rice husks to avoid direct contact. The total protein (TP), lactose, and fat content of colostrum were also analyzed and expressed in percentages using a MilkoScan 104 apparatus (Foss Electric A/S). The IgG concentrations in colostrum were measured with a digital refractometer (PA202X; MISCO). All calves were fed pooled colostrum (protein, mean±standard deviation [SD]: 19.15± 0.34%; fat, 5.10±0.21%; total solid, 30.6±0.04%, IgG>55 g/L) in the amount of 10% of body weight drinking within 1 h post-birth and 2 L of colostrum within 6 h again. Professionals conducted health assessments every day, and finally selected twenty female Holstein heifer calves for the experiment with initial ages (7±0.5 d), body weight (41.65±6.20 kg), and similar body measurement indices.

The experiment utilized a randomized complete block design, grouping calves by their body weight and age, and randomly assigning them to one of two treatments. The control group (n = 10) received the conventional dietary treatment with no additive; The treatment group (n = 10) accepted the routine dietary treatment with 30 g AEPP per head per day. The additive was given to the calves in water-soluble form once a day before drinking milk. During the experiment, calves were offered starter and clean water without restriction. Calves were fed milk replacer (mean±SD: dry matter [DM], 94.56±0.26%, protein, 22.6±0.32%, fat, 16.90±0.18%, ash, 6.82±0.15%, lactose, 40.79±0.12%) in individual mobile plastic bottles (5 L capacity) three times daily (135 g/L, 08:30, 14:30, and 20:30) from 1 to 30 days of trial period (The trial began on the seventh day after birth (d 0) 2.5 L/time from 1 to 7 days of trial period; 3 L/time from 8 to 21 days of trial period; 3.5 L/time from 22 to 30 days of trial period). Table 2 shows the ingredients and nutrient composition of the starter diet. The daily starter intake and refusal by individual calves were recorded, and the starter samples were gathered on d 0 to 2, d 14 to 16, and d 28 to 30 of the experiment.

### Starter and colostrum composition, growth performance, health, blood, rumen fluid and feces collection

Starter samples underwent a preservation process that involved initial heating set to 60°C for 48 hours, subsequent at 105°C for 8 h, and finally being sifted through a 2 mm mesh sieve for storage and subsequent nutritional evaluation. The samples’ DM (AOAC 930.15), crude protein (CP, AOAC 984.13), ether extract (AOAC 920.39), and ash (AOAC 942.05) were measured according to the procedures of AOAC International [12]. Neutral detergent fiber (NDF) and acid detergent fiber (ADF) were analyzed following Hu et al [6]. Starch content was measured by using the related assay kits (product no: K-TSTA; Megazyme International Ireland). The starter intake and rejection of each calf were uniformly documented every day during the duration of the trial. On d 0, 15, and 30, before morning feeding, calves’ body weight was measured with a digital platform scale. Average daily gain (ADG) was computed by determining the daily average change in body weight over each respective period and was divided by the total amount of milk solids and starters consumed to calculate feed efficiency. Additionally, on the same days, measurements of body length, withers height, heart girth, cannon circumference, and hip width were made using a flexible ruler. During measurements, the calves were positioned in a straight state, ensuring relaxed and natural limb positioning without pinching the tail.

Individual health conditions and rectal temperature were examined during the entire experiment period. The basic observations were performed on calves at 08:00 h every day, and the general assessment of daily feces was performed weekly to confirm diarrhea. The 4-point fecal scale method was implemented, with the states of feces gradually changing from normal to watery on a scale of 0 to 3 [7]. When they experienced two successive fecal scores of 2 or a single fecal score of 3 or when they exhibited other symptoms associated with diarrhea, they were subjected to a combination of antibiotic treatments. Abnormal respiratory rate and lung sounds, including wheezing, crackling, pleural friction, or enhanced bronchial sounds, were typical signs that the calf may have suffered bovine respiratory disease [13]. The above operations could also identify all of these diseases in conjunction with a veterinary examination. Throughout the experimental period, calf illnesses were counted, and combinations of gentamicin, penicillin, ampicillin, and cephalosporin were used to treat calf diarrhea and bovine respiratory disease in calves.

On d 0, 15, and 30 of the trial, jugular blood samples were obtained using 10-mL evacuated sodium heparin tubes equipped with disposable blood collection needles before calves were fed in the morning. Subsequently, the samples underwent centrifugation at 2,000×g for a duration of 15 min at a temperature of 4°C. The produced plasma was conserved at −80°C for subsequent examination. The biochemical, antioxidant, and immune metabolic biomarkers were assessed through the utilization of plasma samples in accordance with the procedures detailed in the kits (Jiangsu Meimian Industrial).

Fresh wet fecal samples, each weighing 150 grams, were collected from the rectum at 09:00, 15:00, and 21:00 h on experiment d 0; at 03:00, 06:00, and 18:00 h on experiment d 1; and at 00:00 hours on experiment d 2 [14]. This sampling regimen was repeated during d 14 to 16 and d 28 to 30 of the trial period. Subsequently, the fecal samples were promptly assessed for pH using an electronic Sartorius basic pH meter. Fecal samples weighing 100 g underwent acidification by adding an equal volume of metaphosphoric acid (0.85 mg/L) and then frozen in a −20°C refrigerator until the concentration of each VFA component was determined [15]. On the final day of the experiment, rumen contents were collected from calves via an oral stomach tube prior to morning feeding. Rumen fluid samples were obtained by filtering through four-layered cheesecloth and stored in tubes at −80°C for subsequent analysis.

### Metagenomic and 16S rRNA sequencing analysis

#### Metagenomic DNA extraction, sequencing, and bioinformatics analysis of rumen fluid

Total genomic DNA was extracted from ruminal fluid using the FastPure Soil DNA Isolation Kit (MJYH). DNA concentration, purity, and quality were assessed by TBS-380, NanoDrop2000, and 1% agarose gel. For paired-end sequencing on NovaSeq X Plus (Illumina) at Majorbio Bio-Pharm Technology, DNA was fragmented to 350 bp with Covaris M220 (Gene Company), and libraries were constructed using NEXTFLEX Rapid DNA-Seq (Bioo Scientific).

Raw reads were processed by fastp (v0.23.0) to remove adapters and low-quality sequences (length<50 bp or quality value<20). Host DNA was filtered out by aligning reads to the bovine genome (bosTau8 3.7, https://doi.org/10.18129/B9.bioc.BSgenome.Btaurus.UCSC.bosTau8) using BWA (v0.7.9a). Metagenomic assembly was performed with MEGAHIT (v1.1.2), retaining contigs≥300 bp for further gene prediction and annotation. Open reading frames (ORFs)≥100 bp were predicted using MetaGeneAnnotator and translated with Emboss 6.6.0 and the NCBI translation table into the amino acid sequence. A non-redundant gene catalog was generated using CD-HIT (v4.6.1) with 90% identity and coverage. Gene abundance was then calculated by aligning high-quality reads to this catalog using SOAPaligner (v2.21) at 95% identity.

Non-redundant gene sequences were taxonomically annotated against the NR database and functionally annotated against the Kyoto Encyclopedia of Genes and Genomes (KEGG) database (http://www.genome.jp/kegg/) using Diamond (v0.8.35). Carbohydrate-active enzymes (CAZy) were annotated against the CAZy database (http://www.cazy.org/) using hmmscan ( https://www.ebi.ac.uk/Tools/hmmer/search/hmmscan). All annotations utilized an e-value cutoff of 1e^−5^.

#### Fecal bacterial communities analysis

Total DNA extraction was performed on fecal specimens preserved at −80°C utilizing the E.Z.N.A. DNA Kit (Omega Bio-tek), followed by evaluations of its quality and concentration. Amplification of the 16S rRNA gene’s V3 to V4 region was achieved through using primers 338F (5′-ACTCCTACGGGAGGCAG-3′) and 806R (5′-GGACTACGGGTWTCTAAT-3′). PCR outputs were combined, and DNA underwent purification prior to sequencing using the Illumina MiSeq PE300 system (Shanghai Majorbio Bio-Pharm Technology). Raw sequences were subjected to Fastp quality control, concatenation using Flash, and operational taxonomic units (OTU) clustering with Uparse (97% similarity), along with chimeric sequence removal. The OTUs were then classified using the RDP classifier (a confidence level of 0.7) alongside the Silva 16S rRNA database (v138) for taxonomic annotation. The sequencing raw data generated from this study have been deposited in the National Center for Biotechnology Information Sequence Read Archive under accession number PRJNA1090826.

### Rumen metabolome analysis

The LC–MS/MS analyses of rumen metabolome were performed on the Thermo UHPLC-Q Exactive HF-X system at Majorbio Bio-Pharm Technology. The mobile phase consisted of 0.1% formic acid in water: acetonitrile (95:5, v/v) (solvent A) and 0.1% formic acid in acetonitrile:isopropanol: water (47.5:47.5, v/v) (solvent B). Chromatographic conditions included a flow rate of 0.40 mL/min, column temperature of 40°C, and an injection volume of 5 μL. The UPLC system was coupled to a SCIEX UPLC-Triple TOF 6600 mass spectrometer with an electrospray ionization (ESI) source, operating in both positive and negative modes. Key mass spectrometry parameters were: source temperature 450°C; spray gas 50 psi; Aux gas 13 psi; curtain gas 35 psi; ion-spray voltage −4,500 V (negative) and 5,500 V (positive). MS/MS utilized a 20–40–60 V rolling collision energy. Data were acquired via Data Dependent Acquisition (DDA) mode over a mass range of 50–1,200 m/z.

The data processing were carried out as previously reported [15]. The LC/MS raw data was preprocessed with Progenesis QI software (Waters). Metabolites were identified by searching against the HMDB (http://www.hmdb.ca/), Metlin (https://metlin.scripps.edu/), and the self-compiled Majorbio Database (MJDB) of Majorbio Biotechnology.

### Statistical analysis

All data were tested for normality using the UNIVARIATE procedure in SAS (ver. 9.4; SAS Institute). Growth performance, mean morbidity, plasma metabolites, and fecal fermentation parameters were analyzed using the MIXED procedure. For body measurements, the treatment was the fixed effect, and d 0 data was the covariate for d 15 and d 30 analysis following the model: *Y_ijk_* = *μ*+*T_i_*+*B_j+_C_k_*+*β* (*X̄_ijki_*)+*E_ijk_*, where *Y_ijk_* is the dependent variable, *μ* is the overall mean, *T_i_* is the treatment effect, *B_j_* is the random block effect, *C_k_* is the random effect of calf within treatment, *E_ijk_* is a residual error, *X_ijk_* is covariate of *Y_ijk_*, *X̄_i_* is the mean value of *X_ijk_*, and *b* is the regression coefficient of *Y_ijk_* on *X_ijk_*. Starter intake, ADG, feed efficiency, and antibiotic, administrations/calf were analyzed following the model: *Y_ij_* = *μ*+*T_i_* +*B_j_*+*C_k_*+*E_ij_*, where *Y_ij_* = dependent variable, *μ* = overall mean, *T_i_* = treatment effect, *B_j_* = random block effect, *C_k_* = random effect of calf within treatment, and *E_ij_* = error term. Data on mean fecal score, plasma parameters, and fecal fermentation were analyzed as a randomized complete block design with repeated measures, including the fixed effects of treatment, time, treatment×time interactions, and the random effects of the block and calf within the block. All data from the 0-day time point were included in the model as covariates. The Wilcoxon rank-sum test was used to analyze fecal bacteria, focusing on the relative abundance of bacterial communities at the phylum or genus level in both groups. A p≤0.05 was declared significant, and 0.05<p≤0.10 was declared a trend.

Alpha diversity metrics (Chao1, Ace, Shannon, Simpson) were compared using the Wilcoxon rank-sum test with a false discovery rate (FDR) adjustment (p<0.05) [16]. Microbial community beta diversity, microbiome function, and metabolite levels were assessed by principal component analysis (PCA) based on Euclidean distance, and intergroup differences were evaluated with analysis of similarity (ANOSIM). Partial least squares discriminant analysis (PLS-DA) was used to compare fecal bacterial community structure, and orthogonal projections to latent structures-discriminant analysis (OPLS-DA) was applied to metabolite profiles (R v1.6.2 ropls package) [17]. OPLS-DA model reliability was verified by a 200-permutation test, in which R^2^Y and Q^2^ values close to 1.0 indicated a valid model. Rumen microbial phyla and genera, and the relative abundances of KEGG pathways, KEGG modules, and CAZymes were compared using linear discriminant analysis effect size (LEfSe), with linear discriminant analysis (LDA) score>2 and p<0.05 being considered as significantly different.

For the metabolomics data, positive and negative modes were combined and peak areas normalized. Compounds were annotated via the KEGG database, fold change (FC) between subgroups was calculated, differential significance was assessed by t-test, and variable importance in projection (VIP) from the OPLS-DA model, and differential metabolites were screened with |FC|>1, p<0.05, VIP>1 [17]. The enriched metabolites in KEGG pathways were evaluated by a hypergeometric test, and volcano plots were generated based on the −log10 (p-value) and log2 (FC) of the metabolites, and metabolites of interest were filtered (ggplot2 in R) [15]. The enrichment analysis and random forest analysis were conducted using the online platform MetaboAnalyst 6.0 (https://www.metaboanalyst.ca/). Spearman’s rank correlation was used for all correlation analysis in this study (|R|>0.50 and p<0.05).

## RESULTS

All calves were on normal dietary consumption and no calf deaths occurred throughout the experiment.

### Growth performance

Supplementation with AEPP significantly enhanced the body weight (p = 0.019; p = 0.043) and hip width (p = 0.001; p = 0.007) of calves on d 15 and 30 of the trial period, as depicted in Table 3. Moreover, an increase in withers height (p = 0.020) on d 30 and heart girth (p = 0.032) on d 15 was observed with the administration of AEPP. AEPP treatment also resulted in noticeable improvements in ADG (p = 0.036) and feed efficiency (p = 0.040) of calves throughout d 1 to 30, alongside increased starter intake during d 16–30 (p = 0.042). Notably, there was a trend towards increased starter intake on d 1–30 (p = 0.058) and heart girth on d 30 (p = 0.088) with supplementing AEPP. Nonetheless, there were no notable variances observed in terms of body length and cannon circumference between the control group and the treatment group.

### Mean morbidity

Calves supplemented with AEPP exhibited superior health in comparison to the Control group (Table 4). The Treatment group showed reduced mean morbidity rates of bovine respiratory disease and calf diarrhea and also markedly produced lower administrations/calf of antibiotic therapy (p = 0.040) and improvement of mean fecal scores (p = 0.022).

### Plasma metabolites

As presented in Table 5, the plasma concentrations of albumin (ALB), alanine transaminase (ALT), aspartate transaminase (AST), alkaline phosphatase (ALP), lactate dehydrogenase (LDH), creatine kinase (CK), immunoglobulin M (IgM), interferon-gamma (IFN-γ), haptoglobin (HP), serum amyloid A (SAA), and catalase (CAT) activity between the control and the treatment groups were not significantly different. However, the treatment group calves exhibited significantly higher concentrations of plasma glucose (GLU, p = 0.013), TP (p = 0.045), globulin (GLB, p = 0.022), immunoglobulin A (IgA, p<0.001), immunoglobulin G (IgG, p<0.001), interleukin-10 (IL-10, p = 0.021) when compared to control group. Conversely, plasma malondialdehyde (MDA, p<0.001), tumor necrosis factor-alpha (TNF-α, p = 0.047), interleukin-1-beta (IL-1β, p = 0.033), and interleukin-6 (IL-6, p<0.001) concentrations were lower in the treatment group calves. Additionally, the treatment group exhibited significantly enhanced levels of total antioxidant capacity (T-AOC, p<0.001) and, glutathione peroxidase (GSH-Px, p<0.001) and total superoxide dismutase (T-SOD, p = 0.002) activities relative to the control group.

### Rumen microbiome composition and taxonomic differences

**Figure 1:**
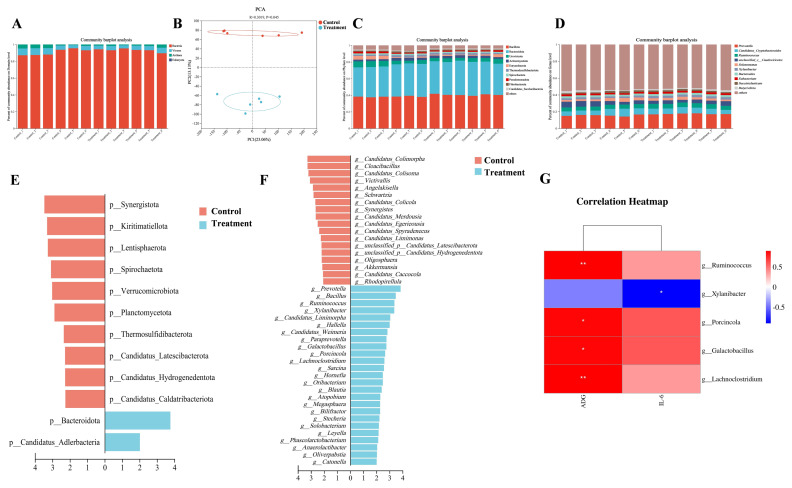
Analysis of rumen microbiota in the control group and the treatment group. (A) Relative abundance (%) of rumen microbial domains in calves. (B) Principal component analysis (PCA) plot of calves based on rumen bacterial species-level composition. (C) Relative abundance of the top 10 predominant rumen bacterial phyla in calves, and categorization of other low abundance species as others. (D) Relative abundance of the top 10 predominant rumen bacterial genera in calves, and categorization of other low abundance species as others. (E) Linear discriminant analysis effect size (LEfSe) identifying differentially abundance of rumen bacterial phyla (linear discriminant analysis [LDA]>2, p<0.05). (F) LEfSe analysis identifying differentially abundance of rumen bacterial genera (LDA>2, p<0.05). (G) Correlation heatmap between the relative abundance of rumen bacterial genera and calf phenotypic indicators. Significant correlations are defined as |R|>0.5 and p<0.05, with red and blue colors indicating positive and negative correlations, respectively. * p<0.05, ** p<0.01.

A total of 507,029,142 raw reads were generated from rumen metagenomic sequencing. Each sample yielded an average of 42,252,429±2,264,509 reads (mean±standard error of the mean). After quality control and host gene removal, 480,468,510 reads remained, averaging 40,039,043±2,268,999 reads per sample. Subsequently, contig assembly resulted in 3,014,397 contigs in total, with an average of 251,200±14,470 contigs per sample (Supplement 1). The relative abundance of microbial domains within the rumen microbiome of the two groups included Bacteria (91.96%), Viruses (5.63%), Archaea (2.39%), and Eukaryota (0.01%), with Bacteria being the predominant domain (Figure 1A; Supplement 2). Alpha diversity analysis of bacterial communities revealed significant differences in Shannon and Simpson indices between the two groups (p<0.05), and a trend towards differences in Ace and Chao1 indices (p = 0.089), indicating significant differences in rumen bacterial richness and diversity between the control group and the treatment group (Supplement 3). Furthermore, PCA showed a clear separation between the control group and the treatment group at the bacterial species level (p<0.05) (Figure 1B). Based on the observed differences, subsequent analyses primarily focused on comparing bacterial taxonomy in the rumen of calves between the control group and the treatment group. At the phylum level, the rumen microbiomes of both the treatment group and the control group calves were predominantly composed of Bacillota (39.36%), Bacteroidota (38.63%), and Uroviricota (4.99%). At the genus level, the dominant genera included *Prevotella* (16.39%), *Candidatus_Cryptobacteroides* (6.67%), *Ruminococcus* (5.09%), *unclassified_c__Caudoviricetes* (4.80%), *Selenomonas* (2.87%), and *Xylanibacter* (2.43%) (Figure 1C, 1D; Supplement 4). Further evaluation of bacterial differences at the phylum and genus levels was conducted using LEfSe analysis. The rumen bacteria of the treatment group calves exhibited enrichment of Bacteroidota and Candidatus_Adlerbacteria at the phylum level, along with 25 additional bacterial genera enrichment (LDA>2, p<0.05; Figure 1E, 1F, Supplement 5). The enrichment of these taxa within the rumen bacterial community of the treatment group suggested a potentially more active metabolic capacity in the rumen microbiota of these calves.

Correlation analysis was performed between rumen bacterial genera enriched in the treatment group and calf growth performance and health indicators. Results revealed that *Ruminococcus*, *Lachnoclostridium*, *Galactobacillus*, and *Porcincola* were positively correlated with ADG, while *Xylanibacter* was negatively correlated with IL-6 (|R|>0.5, p<0.05; Figure 1G, Supplement 6). These findings were classified as potential rumen-associated microorganisms relevant to calf performance and health.

### Functional profile and differential analysis of rumen microbiome

**Figure 2:**
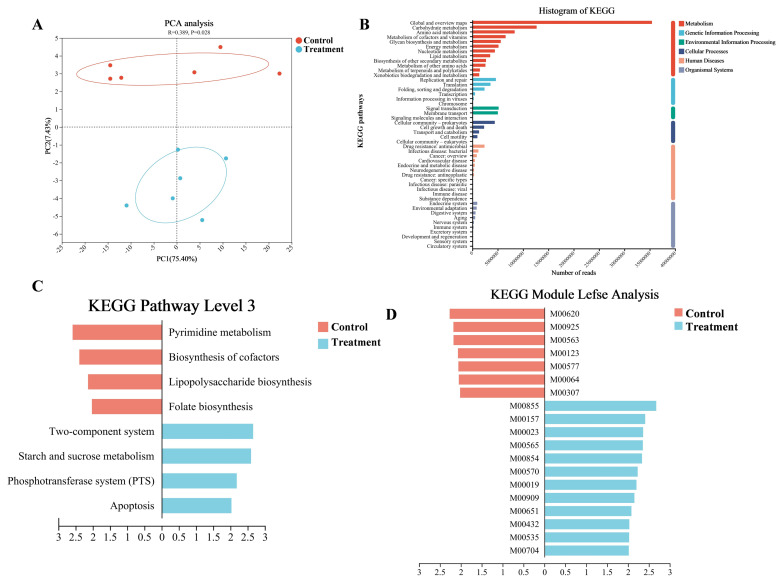
Differences in Kyoto Encyclopedia of Genes and Genomes (KEGG) functional capacities of the rumen microbiome between the control and the treatment groups. (A) Principal component analysis (PCA) plot based on KEGG level 3 pathways. (B) KEGG histogram based on pathway level 1 and pathway level 2. (C,D) Linear discriminant analysis effect size (LEfSe) identifying differentially abundance of KEGG level 3 pathways (C) and functional modules (D), with statistical significance being defined as linear discriminant analysis (LDA)>2 and p<0.05.

Consistent with the observed differences in rumen microbial communities, the rumen microbial functions in the treatment group were also significantly altered compared to the control group. Functional analysis was conducted using the KEGG and CAZymes profiles. PCA based on KEGG level 3 pathways revealed a clear separation between the two groups of calves (p<0.05; Figure 2A). Categorization of KEGG pathways revealed that the predominant functions in both groups were associated with the level 1 pathway “Metabolism”. Within this category, the most abundant level 2 pathways were “Global and overview maps”, “Carbohydrate metabolism”, and “Amino acid metabolism” (Figure 2B). To identify differences in KEGG level 3 pathways between the two groups, LEfSe analysis was performed. Compared to the control group, four pathways, including “Starch and sucrose metabolism”, were enriched in the rumen of the treatment group calves (LDA>2, p<0.05; Figure 2C, Supplement 7). Furthermore, a comparison of KEGG modules between the two groups revealed that 12 modules were enriched in the treatment group, including “Trehalose biosynthesis, D-glucose 1P => trehalose,” “Glycogen degradation, glycogen => glucose-6P,” and “Glycogen biosynthesis, glucose-1P => glycogen/starch,” all of which belong to the “Starch and sucrose metabolism” pathway (LDA>2, p<0.05; Figure 2D, Supplement 8).

**Figure 3:**
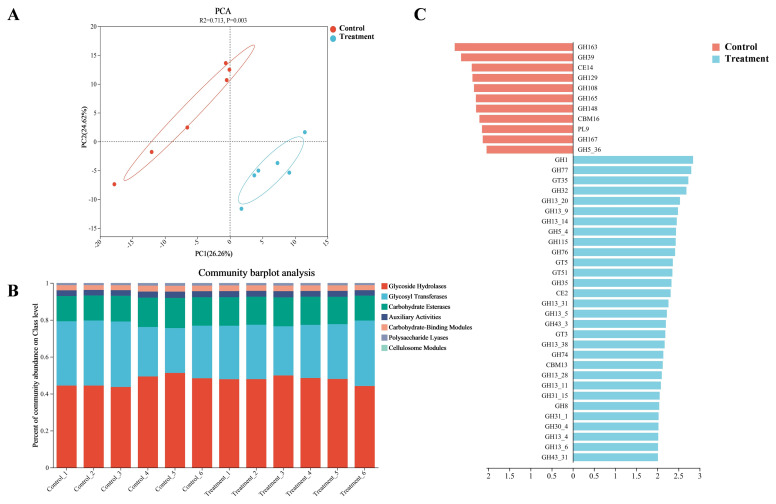
Comparison of carbohydrate-active enzymes (CAZymes) profiles in the rumen microbiome between the control and the treatment groups. (A) Principal component analysis (PCA) plot based on CAZymes family-level annotations. (B) The relative abundance (%) of rumen CAZymes-class level in calves. (C) Linear discriminant analysis effect size (LEfSe) identifying differentially abundance of CAZymes-family level (linear discriminant analysis [LDA]>2, p<0.05).

To further investigate carbohydrate metabolism within the calf rumen, the CAZymes profile was utilized to analyze the rumen microbiomes of both groups. PCA at the family level showed a clear separation between the two groups (p<0.05; Figure 3A). The composition at the class level primarily consisted of glycoside hydrolases (GHs, 47.44%), glycosyl transferases (GTs, 30.17%), carbohydrate esterases (CEs, 14.94%), auxiliary activities (AAs, 3.19%), carbohydrate-binding modules (CBMs, 2.94%), and polysaccharide lyases (PLs, 1.26%), with cellulosome modules (CMs, 0.05%; Figure 3B, Supplement 9). Within the rumen microbiome of the treatment group, 24 GHs, 4 GTs, 1 CBM, and 1 CEs were enriched (LDA>2, p<0.05; Figure 3C, Supplement 10), suggesting an enhanced potential for glycosidic bond hydrolysis within the rumen microbiome of the treatment group.

### Differences in the rumen metabolome profile of calves

**Figure 4:**
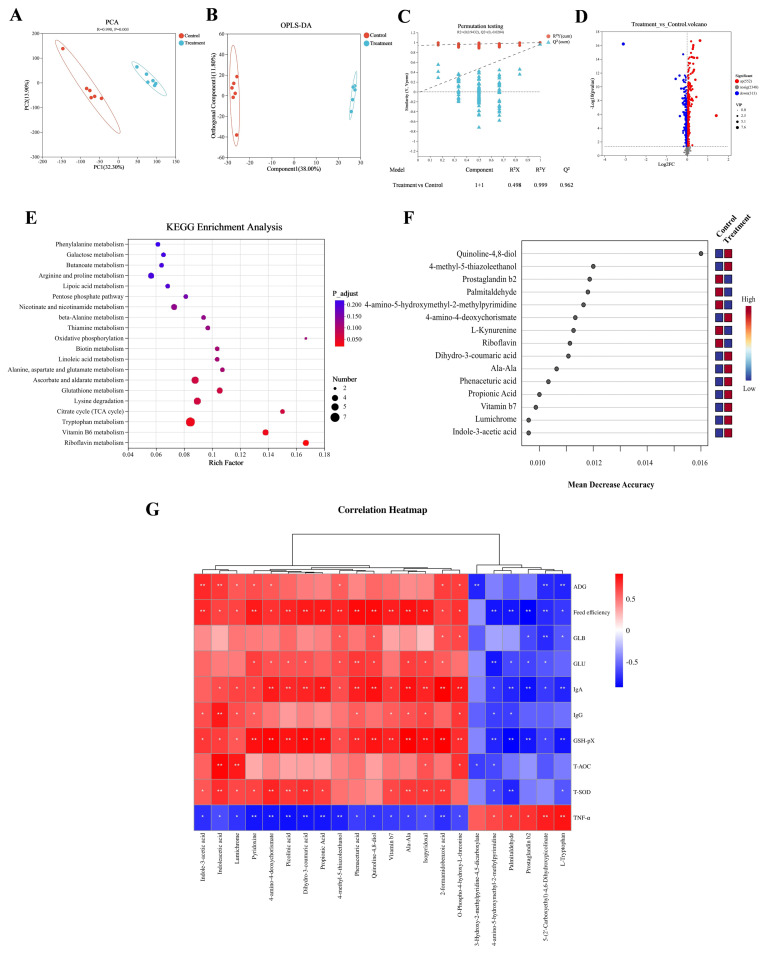
Enrichment of rumen metabolites and their relationship with host phenotypic indicators. (A) Principal component analysis (PCA) plot comparing the overall rumen metabolite profiles. (B) The orthogonal projections to latent structures-discriminant analysis (OPLS-DA) plot. (C) Permutation test for OPLS-DA, with R^2^Y and Q^2^Y indicating model interpretability and predictability, respectively. (D) Volcano plot depicting the overall trend of differential rumen metabolite content between the control and the treatment groups. (E) Kyoto Encyclopedia of Genes and Genomes (KEGG) pathway enrichment analysis based on significantly different rumen metabolites between the control and the treatment groups. (F) Top 15 rumen metabolites identified using random forest. The difference in the color of the two groups of boxes in the same row reflects the difference in the relative abundance of the metabolite between the groups, with red and blue representing higher or lower abundances, respectively. (G) Correlation heatmap between rumen metabolites and host phenotypic indicators. Significant correlations are defined as |R|>0.5 and p<0.05, with red and blue colors indicating positive and negative correlations, respectively. * p<0.05, ** p<0.01. ADG, average daily gain; GLB, globulin; GLU, glucose; IgA, immunoglobulin A; IgG, immunoglobulin G; GSH-Px, glutathione peroxidase; T-AOC, total antioxidant capacity; T-SOD, total superoxide dismutase; TNF-α, tumor necrosis factor-α.

PCA plot revealed a distinct separation in metabolic profiles between the control group and the treatment group (Figure 4A). The OPLS-DA plot, validated by 100 permutation tests, confirmed that the model was robust and not overfitted (Q^2^ intercept<0, R^2^Y = 0.999, Q^2^ = 0.962; Figure 4B, 4C). Comparative analysis using t-tests and VIP identified 865 differentially metabolites, including 552 upregulated and 313 downregulated metabolites (VIP>1, p<0.05; Figure 4D). Enrichment analysis of these differential metabolites using the KEGG database revealed enrichment in pathways such as “Tryptophan metabolism”, “Vitamin B6 metabolism”, and “Riboflavin metabolism” (p<0.05; Figure 4E; Supplement 11), with specific metabolites like indoleacetic acid, picolinic acid, and 5-(2′-Carboxyethyl)-4,6-Dihydroxypicolinate being annotated to these pathways (Supplement 12). Concurrently, a random forest analysis identified the top 15 key metabolites, each with a mean decrease in accuracy (MDA) value≥0.1, which included quinoline-4,8-diol, 4-methyl-5-thiazoleethanol, prostaglandin b2, palmitaldehyde, 4-amino-5-hydroxymethyl-2-methylpyrimidine, 4-amino-4-deoxychorismate, L-kynurenine, riboflavin, dihydro-3-coumaric acid, ala-ala, phenaceturic acid, and propionic acid (Figure 4F; Supplement 13). Subsequently, a correlation analysis was conducted to investigate the associations between two types of metabolites (those associated with significantly enriched KEGG pathways and key metabolites identified by the random forest analysis) and calf phenotypic indicators (related to growth performance and health) (Figure 4G; Supplement 14). This analysis showed significant associations between metabolites and growth indicators, antioxidant factors, and immunoglobulins. Specifically, 16 metabolites, including indoleacetic acid and picolinic acid, were significantly elevated in the Treatment group and exhibited positive correlations with ADG, feed efficiency, GSH-Px, T-SOD, T-AOC, IgA, and IgG. Furthermore, six metabolites, also significantly elevated in the treatment group, were negatively correlated with the pro-inflammatory factor TNF-α (|*R*|>0.5, p<0.05).

### Correlation analysis of the rumen microbiome and metabolome

**Figure 5:**
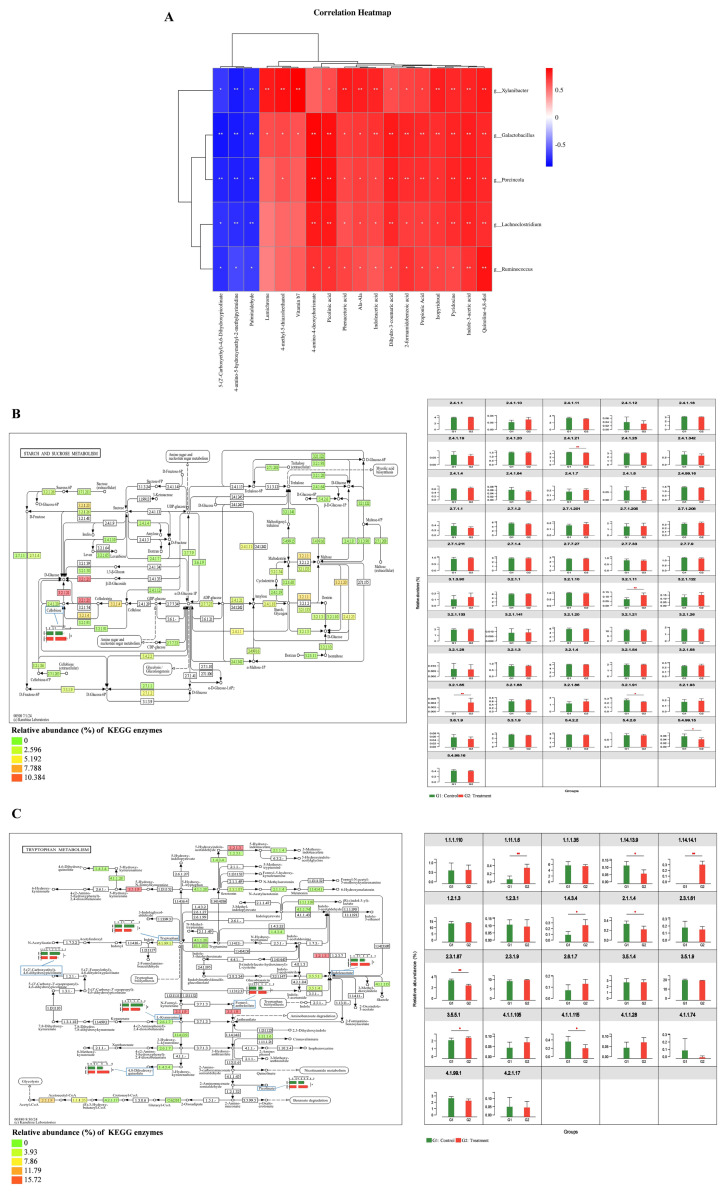
Relationship between the rumen-associated microbiome and metabolome. (A) Correlation heatmap of rumen bacterial genera and metabolites linked to host phenotypic indicators. Significant correlations are defined as |R|>0.5 and p<0.05, with red and blue colors indicating positive and negative correlations, respectively. (B,C) Differentially enriched metabolic pathways. Each box represents a sample group, and the color intensity reflects the relative abundance of enzymes. The bar chart in the left panel shows the comparison of differential metabolite abundance, while the bar chart in the right panel displays the comparison of enzyme abundance between the control and the treatment groups. * p<0.05, ** p<0.01.

Investigation into the relationships between identified rumen-associated microorganisms and the aforementioned related metabolites revealed significant correlations (Figure 5A; Supplement 15). Specifically, *Galactobacillus*, *Lachnoclostridium*, *Porcincola*, *Ruminococcus*, and *Xylanibacter* were positively correlated with metabolites associated with tryptophan metabolism (quinoline-4,8-diol, 2-formamidobenzoic acid, picolinic acid, indoleacetic acid, and indole-3-acetic acid), vitamin B6 metabolism (pyridoxine and isopyridoxal), as well as ala-ala, phenaceturic acid, propionic acid, and dihydro-3-coumaric acid (|*R*|>0.5, p<0.05). In contrast, these microbial genera were negatively correlated with three metabolites that decreased in the treatment group: 5-(2′-carboxyethyl)-4,6-dihydroxypicolinate (a tryptophan metabolism-related metabolite), palmitaldehyde, and 4-amino-5-hydroxymethyl-2-methylpyrimidine (|*R*|>0.5, p<0.05). To further elucidate the activation status of metabolic pathways in the calf rumen, the relative abundances of key KEGG enzymes and metabolites involved in the Starch and sucrose metabolism and Tryptophan metabolism pathways were examined (Figure 5B, 5C).

### Fecal fermentation

Oral administration of AEPP throughout the trial period demonstrated a positive impact on fecal fermentation parameters, as indicated in Table 6. The treatment group exhibited a higher concentration of total VFA (p<0.001) and a greater butyrate molar proportion (p<0.001) throughout the entire trial duration. Conversely, fecal pH (p<0.001) in calves receiving AEPP supplementation decreased significantly than that of calves without additives. However, feeding AEPP yielded a nonsignificant impact on the molar proportion of VFA (p> 0.05), except for butyrate.

### Bacterial communities

**Figure 6:**
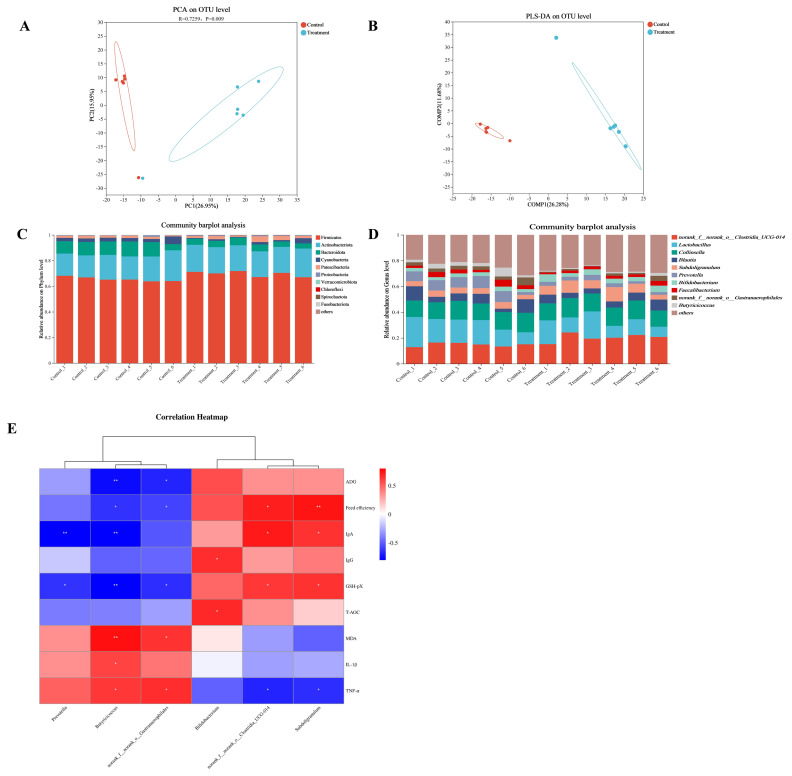
Differences in bacteria abundance in the feces of calves between the control group and the treatment group and the relationships between fecal microbiome and host phenotypic indicators. (A) Principal component analysis (PCA) plot showing the comparison of the overall bacterial microbiota. (B) Partial least squares discriminant analysis (PLS-DA) of bacterial community structure. (C,D) Composition of the top 10 predominant rumen bacterial phyla and genera in calves, and categorization of other low abundance species as others. (E) Correlation heatmap between bacterial genera in feces and host phenotypic indicators. Significant correlations are defined as |R|>0.5 and p<0.05, with red and blue colors indicating positive and negative correlations, respectively. * p<0.05, ** p<0.01. OTU, operational taxonomic unit; ADG, average daily gain; IgA, immunoglobulin A; IgG, immunoglobulin G; GSH-Px, glutathione peroxidase; T-AOC, total antioxidant capacity; MDA, malondialdehyde; IL-1β, interleukin-1β; TNF-α, tumor necrosis factor-α.

The Alpha diversity indices of bacteria in the feces of calves are presented in Supplement 16. ACE (p = 0.001), Chao 1 (p = 0.001), and Shannon (p = 0.001) were elevated, while the Simpson (p = 0.037) was reduced in the Treatment group. Furthermore, the supplementation of AEPP was noted to significantly alter the fecal microbial proportion in calves, influencing the relative abundance of diverse bacteria. Moreover, PCA and PLS-DA analyses were employed to assess the bacterial composition in the feces of calves (Figure 6A, 6B). The PCA-PC1 and PCA-PC2 axes accounted for 26.95% and 15.95% of the total variance, and the PLS-DA-COMP1 and PLS-DA-COMP2 axes accounted for 26.28% and 11.68% of total variance respectively. The results revealed a substantial difference in bacteria abundance for AEPP to add or not, with a notable separation observed between the control and treatment groups. The discoveries on the 10 most predominant bacteria at the phylum and genus level in calf feces, highlighting AEPP’s impact on the relative abundance of the bacterial population (Figure 6C, 6D). The composition between the bacteria phyla of the two groups exhibited noteworthy changes (Supplement 17). The abundances of Firmicutes (p = 0.010) and Chloroflexi (p = 0.004) enhanced with AEPP supplementation, and the relative abundance of Actinobacteriota showed an upward tendency (p = 0.055), whereas those of Bacteroidota (p = 0.025), Cyanobacteria (p = 0.037), Verrucomicrobiota (p = 0.016), and Fusobacteriota (p = 0.007) reduced significantly. AEPP supplementation notably elevated the relative abundance of *norank_f__norank_o__Clostridia_UCG-014* (p = 0.010), *Subdoligranulum* (p = 0.037), and *Bifidobacterium* (p = 0.025). In contrast, it lowered *Prevotella* (p = 0.025), *norank_f__norank_o__Gastranaerophilales* (p = 0.037), and *Butyricicoccus* (p = 0.010) in feces. Correlation analysis was performed between the top 10 most abundant bacterial taxa and calf growth performance and health-related phenotypes. In the treatment group, higher relative abundances of *norank_f__norank_o__Clostridia_UCG-014* and *Subdoligranulum* were positively correlated with feed efficiency and antioxidant factors such as GSH-Px, while negatively correlated with the pro-inflammatory factor TNF-α. Furthermore, lower abundances of *Butyricicoccus* and *norank_f__norank_o__Gastranaerophilales* in the Treatment group were negatively correlated with feed efficiency and antioxidant factors but positively correlated with TNF-α, showing a pattern opposite to the aforementioned (|*R*|>0.5, p<0.05; Figure 6E, Supplement 18).

## DISCUSSION

Previous studies have demonstrated that supplementation with probiotics or edible plants can improve calf health and promote growth [6,7]. In this experiment, a co-fermented combination of probiotics and edible plants was selected as a feed additive, and the results showed that AEPP was able to improve growth performance, disease resistance, antioxidant function, plasma and rumen metabolites, and fecal fermentation parameters and maintain a positive balance of the rumen and hindgut microbiota, promoting the health and growth of pre-weaned calves.

Including additives in the calf diet led to notable enhancements in starter intake and weight gain. In *T. chebula* and *C. setosum* (Willd.) MB, flavonoids, and triterpenes stand out as the key bioactive substances. The biological impacts have probably been crucial in fostering calf growth. Flavonoids have been observed to modulate the release of growth hormone. This mechanism facilitates accelerated bone formation, thereby fostering the growth of the body frame in calves [11]. However, in this experiment, AEPP was able to exert good biological effects even at a lower intake of total flavonoids and triterpenes, which might also be related to the synergistic effect of probiotics. *S. cerevisiae* in AEPP can stimulate the rumen to decompose cellulose and promote the utilization of lactic acid through the metabolism of minerals, organic acids, or vitamins, improve the energy utilization of calves [9], and help to ensure the rumen health of calves under the condition of high starter intake. The cellulase secreted by *B. subtilis* also helps break down cellulose and other complex carbohydrates, improving palatability and promoting increased intake of starters [8].

The use of antibiotics for disease treatment can adversely affect the gastrointestinal microbiota in calves, significantly impacting milk production during the initial lactation [1,18]. Ongoing studies focus on choosing natural plants as substitutes to lessen the adverse impacts linked to the use of antibiotics. *T. chebula* and *C. setosum* (Willd.) MB has demonstrated efficacy in protecting epithelial cells against influenza virus invasion, lowering the prevalence of diarrhea and respiratory diseases, and the bioactive constituents (flavonoids and triterpenes) contained showed a crucial function in them. It has been demonstrated that the supplementation with these edible plants reduced the frequency of respiratory disease and diarrhea in calves by inhibiting pathogenic bacteria [4,5]. Moreover, the cell wall components of *S. cerevisiae* contain oligosaccharides that adhere to pathogens and reduce their contact with the intestinal mucosa, alleviating the risk of diarrhea [9]. *B. subtilis* forms biofilm bunches with probiotics such as lactic acid bacteria to protect them from pathogens by secreting lipopeptides with antibacterial activity or inducing the expression of tapA operons associated with protective extracellular matrix [19]. Supplementing *L. plantarum* can secrete lactic acid, reduce intestinal pH, and impede the pathogenic microorganisms’ growth, thus lowering morbidity and improving growth performance in young ruminants [7]. The bioactive ingredients in AEPP have a stacking effect and work together to promote calf health.

The heightened GLU levels in AEPP-supplementation calves suggested an enhanced efficiency of energy metabolism in calves and improved body development, which might be attributed to the fact that increased starter intake provides more digestive material in the body of calves. The levels of TP can reflect whether passive immunity is initiated after colostrum intake, and low levels of TP are strongly associated with high calf death rates [9]. In this experiment, the high TP in the Treatment group indicated a positive effect of AEPP on calf immunity. The GLB reflects the amino acid levels obtained by the calves from the diet, and an increase in its content might be associated with increased immunoglobulin concentrations in the treatment group. According to reports, *B. subtilis* and *Lactobacillus* interact with the microbial community resident in the gastrointestinal, epithelium, and immune cells to initiate and facilitate immune function, resulting in the release of antibodies [7,19]. The presence of probiotics in the AEPP markedly increased the calves’ IgA and IgG concentrations, enhancing their immune response and aiding in the reduction of the inflammatory reaction of the organism. Meanwhile, probiotics and flavonoids in the additive inhibited the production of pro-inflammatory factors TNF-α, IL-1β, and IL-6 and promoted the production of the anti-inflammatory factor IL-10, which together diminish inflammation [20,21]. Antioxidant function is an important measure of the health status of animals. The supplementation of probiotics, flavonoids, and triterpenoid compounds in animal diets has been shown to positively regulate the activities of T-AOC, T-SOD, and GSH-Px [6,9]. Enhanced antioxidant capacity in the serum of the treatment group calves can more effectively neutralize free radicals and alleviate oxidative stress, thereby enhancing the body’s disease resistance, mitigating the deleterious effects of diseases on growth delays, and promoting better growth performance in the animals.

Bacteria constitute the dominant microbial domain in the calf rumen, playing a pivotal role in promoting digestive and metabolic processes. Although fewer differentially enriched phyla, namely Bacteroidota and *Candidatus_Adlerbacteria*, were observed in the treatment group compared to the control group, the treatment group exhibited a higher number of genera with elevated relative abundances, including *Prevotella*, *Galactobacillus*, *Ruminococcus*, and *Xylanibacter*, thereby promoting digestive and metabolic processes within the rumen. The AEPP facilitated increased starter feed intake in the treatment group calves. Consistent with this, the Treatment group showed notably higher abundances of these fiber-degrading bacteria such as *Prevotella*, *Ruminococcus*, and *Xylanibacter* [3,22], as well as propionic acid abundance and feed efficiency, compared to the control group. Carbohydrates are decomposed by fiber-degrading bacteria to produce short-chain fatty acids (SCFA) like propionic acid, and a positive correlation was observed between *Ruminococcus*, *Xylanibacter*, and propionic acid. Furthermore, propionic acid was remarkably positively correlated with feed efficiency. Collectively, these results suggested that AEPP improved feed utilization and growth performance in calves by enhancing starter intake, which in turn increased the abundance of fiber-degrading bacteria and boosted the efficiency of carbohydrate conversion into propionic acid.

Similarly, *Xylanibacter* can decompose carbohydrates to produce SCFAs [22]. SCFAs can inhibit pro-inflammatory factors. In the treatment group, *Xylanibacter* was negatively correlated with IL-6, which suggested that *Xylanibacter* might possess the ability to modulate host inflammatory responses. *Galactobacillus*, along with the *L. plantarum* in AEPP, belongs to lactic acid bacteria, which can produce lactic acid, maintain the ruminal environment, and promote the proliferation and metabolic activity of rumen microorganisms [7]. Likewise, *Lachnoclostridium*, a member of the Lachnospiraceae family, is capable of utilizing carbohydrates to generate SCFAs, and positive correlations between other genera within the Lachnospiraceae family and propionic acid have been reported in other studies [15]. Therefore, its increased abundance in the Treatment group likely contributed to the elevated propionic acid abundance.

Rumen microorganisms are central to the functions within the rumen, and indeed, most KEGG functions demonstrated higher activity in the treatment group. Specifically, the carbohydrate metabolism-related pathway “Starch and sucrose metabolism” was significantly enriched in the treatment group, indicating an enhanced capacity for carbohydrate degradation in the rumen of the treatment group calves and potentially leading to increased abundances of degradation products such as Cellobiose. Furthermore, the treatment group exhibited a greater abundance of carbohydrate-degrading enzymes, including 24 GHs and 1 CE, collectively facilitating the carbohydrate metabolic processes within the calf rumen.

The interplay between metabolites and microorganisms influences calf phenotypic indicators. In the treatment group, differentially abundant metabolites in the calf rumen were primarily enriched in the tryptophan metabolism and vitamin B6 metabolism pathways. Tryptophan metabolism dysregulation was closely associated with inflammatory responses. Specifically, L-Kynurenine is known to promote the increase of the pro-inflammatory factor TNF-α by activating relevant receptors, leading to promotion of inflammation and oxidative stress [23]. The decreased abundance of L-Kynurenine observed in the Treatment group might be attributed to its metabolic breakdown into products such as quinoline-4,8-diol and picolinic acid. The reduction in abundance of L-Kynurenine, in turn, was associated with lower TNF-α levels and decreased oxidative stress. The negative correlation observed in this study between quinoline-4,8-diol and picolinic acid with TNF-α, as well as their positive correlation with antioxidant indicators like GSH-Px, further supports this interpretation. Additionally, *Lactobacillus* and *Ruminococcus* possessed the ability to metabolize tryptophan into indole and its derivatives (e.g., indoleacetic acid and indole-3-acetic acid) [23]. These derivatives were recognized for their roles in regulating inflammation, antioxidant activity, and the immune system, thereby helping to suppress the increase of pro-inflammatory factors [23]. The enrichment of *Galactobacillus* and *Ruminococcus* genera in the Treatment group facilitated the breakdown of tryptophan into indoleacetic acid and indole-3-acetic acid. In this study, the abundances of *Galactobacillus* and *Ruminococcus* showed positive correlations with indoleacetic acid and indole-3-acetic acid. Moreover, these same metabolites were positively correlated with IgG and antioxidant factors (GSH-Px and T-SOD) but negatively correlated with TNF-α. These findings suggested that *Galactobacillus* and *Ruminococcus* might collectively contribute to improving calf health by modulating tryptophan metabolism to mitigate inflammatory responses, enhance immune function, and bolster antioxidant capacity.

B vitamins, as essential coenzymes for various enzymes, are extensively involved in carbohydrate, lipid, protein, and nucleic acid metabolism [17]. In this study, upregulated differential metabolites in Vitamin B6 metabolism (pyridoxine, isopyridoxal, and o-phospho-4-hydroxy-L-threonine), as well as riboflavin metabolism (Lumichrome), showed significant positive correlations with ADG or feed efficiency. This finding suggested that the enhanced activity of these two B vitamin metabolic pathways contributed to the improved metabolic processes observed in the treatment group. Elevated abundances of the amino acid metabolism intermediates phenaceturic acid and ala-ala in the treatment group indicated heightened protein metabolic activity. The enhanced pathway activity likely promoted microbial protein synthesis and improved feed utilization efficiency [24]. In summary, the aforementioned functional rumen microorganisms and differential metabolites likely exert synergistically to promote calf growth, enhance antioxidant capacity, and mitigate inflammatory responses.

The increase in hindgut SCFA concentrations can be attributed to many factors. *Bifidobacterium* can promote the addition of SCFA and decrease pH by competing for intestinal epithelial attachment sites and nutrients and secreting bacteriocin [25]. Therefore, the supplementation of AEPP in this study promoted the increase of the relative abundance of *Bifidobacterium*, which might be one of the ways to improve the concentration and composition of intestinal SCFA in calves. Butyrate acted as a preferential energy source for the colon and improved the intestinal epithelial cell barrier and permeability to resist intestinal invasion of pathogens for calves [9]. *L. plantarum* can produce organic acids such as propionic, lactic, acetic, and butyric acid and promote the reduction of fecal pH [7]. Furthermore, the modulation of the relative abundance of butyric acid-producing bacteria by AEPP in this study might underlie the observed increase in butyric acid content. The soluble carbohydrates undergo hydrolysis into glucose by digestive enzymes. *Lactobacillus* and *Bifidobacterium*, which are capable of producing lactic acid, convert a fraction of glucose to organic acid. Subsequently, glycolysis and flavin cross mechanisms collaboratively promote pyruvate production and, ultimately, through the butyryl-CoA: acetate CoA-transferase pathway, contribute to the synthesis of butyric acid [26]. However, the complex genes that play a catalytic role in the pathway are usually present in butyric acid-producing bacteria (*Subdoligranulum* and *Collinsella*) of Firmicutes [27]. In addition to the above-mentioned butyric acid production pathways, we also speculate that the bioactive components of edible plants in AEPP might also play a role in improving butyric acid production. Madecassoside (triterpenoid extract) can increase butyric acid composition in animal feces through the augmentation of the butyric acid-producing bacterial population [28], suggesting a remarkable role for AEPP in promoting the production of SCFA in the intestinal tract of calves and in maintaining intestinal health.

Comparative analysis with the control group showed that applying AEPP exerted a beneficial influence on the relative abundance, diversity, and community composition of fecal bacteria, as evidenced by alterations in Alpha and Beta indices. These findings suggested a notable gut-microbiota restructuring in ruminants, potentially linked to the functional regulation or relevant factors associated with the probiotics and bioactive compounds present in AEPP. Furthermore, the heightened richness and diversity observed in bacterial communities within the treatment group might be linked to reduced antibiotic interference in calves. Triterpenoids were found to influence *norank_f_norank_o_Clostridia_UCG-014* abundance, contributing to the maintenance of intestinal flora homeostasis, the multiplication of beneficial bacteria, and the improvement of nutrient digestion, absorption, and immunity [29]. In this study, *norank_f_norank_o_Clostridia_UCG-014* was positively correlated with ADG and feed efficiency, and negatively correlated with TNF-α level. These correlations suggested that the bacteria might enhance nutrient absorption and regulate inflammatory factors. Furthermore, a significant negative correlation was observed between the relative abundance of *Subdoligranulum* and TNF-α level. This finding was consistent with previous reports indicating a positive correlation between *Subdoligranulum* and fecal butyrate production [27], a metabolite known for its anti-inflammatory properties and role in maintaining gut homeostasis [9]. These data suggested that *Subdoligranulum* might contribute to the alleviation of inflammation through butyrate-related pathways. Additionally, it has been reported that there was an inverse relationship between the relative abundance of *Butyricicoccus* and the intake of lactic acid, suggesting that a reduction in its relative quantity could boost carbohydrate absorption [30]. Probiotics in AEPP demonstrated excellent carbohydrate fermentation capabilities in the hindgut, facilitating the utilization of nutrients from the starter consumed by calves while producing organic acids and promoting butyrate production [7,9]. Following the supplementation of AEPP, the relative abundance of *Prevotella*, which belonged to saccharolytic ability taxa [22], might decrease due to competitive inhibition with *L. plantarum* for substrates, potentially explaining its reduced relative abundance. In summary, AEPP contributed to the improvement of the health and growth performance of preweaned calves by structurally reshaping the gastrointestinal microbiota, stimulating the generation of beneficial metabolites, and improving antioxidant capacity.

## CONCLUSION

This study demonstrated that dietary supplementation with AEPP beneficially modulated the rumen and hindgut microbiota, leading to favorable shifts in metabolite profiles and activation of key metabolic pathways. Furthermore, AEPP supplementation also enhanced the antioxidant capacity of calves. These changes collectively contributed to improved growth performance, enhanced immune function, and reduced disease incidence in pre-weaned calves, confirming AEPP as a safe and effective dietary strategy and a viable alternative to antibiotics for pre-weaning calves during their susceptible stage. These findings provide a reference for future studies on the health and nutritional regulation of young ruminants. Whether feeding AEPP in the complete growth stage of calves can affect the changes of ruminal and intestinal microflora and exert the same effect of promoting growth and reducing morbidity needs to be further verified.

## Figures and Tables

**Table 1:** The main content of plant secondary metabolites and the strain number of live probiotics in the additives from co-fermented with edible plants and probiotics

Item	AEPP
The content of plant secondary metabolites (mg/g)	
Total flavonoids (as rutin equivalents)	1.03
Total triterpenes	3.43
Strain number of live probiotics (CFU/g)	
*Saccharomyces cerevisiae*	7.50×10^8^
*Lactobacillus plantarum*	5.63×10^8^
*Bacillus subtilis*	4.50×10^8^

AEPP, the additives from co-fermented with edible plants and probiotics.

**Table 2:** Ingredients and nutrient composition of the calf starter

Item	Starter diet, % (DM basis)
Ingredient	
Ground corn	39.00
Extruded soybean	9.50
DDGS	4.00
Soybean meal	27.00
Soybean hulls	6.60
Wheat bran	6.60
Molasses	3.00
Limestone	1.50
Sodium bicarbonate	0.40
Sodium chloride	0.80
Magnesium oxide	0.20
Calcium hydrogen phosphate	0.40
Premix^1)^	1.00
Chemical composition	
DM	90.33
CP	23.48
Starch	32.80
Ether extract	4.75
NDF	18.10
ADF	9.50
Ash	8.59

1) Premix supplied the following nutrients per kilogram of mixed feed: vitamin A 6,500 IU, vitamin D 2,000 IU, vitamin E 120 IU, Fe 80 mg, Co 0.3 mg, Cu 16 mg, I 1.1 mg, Mn 50 mg, Se 0.45 mg, and Zn 70 mg.

DM, dry matter; DDGS, distillers dried grains with solubles; CP, crude protein; NDF, neutral detergent fiber; ADF, acid detergent fiber.

**Table 3:** Effect of additives from co-fermented with edible plants and probiotics on growth performance of Holstein calves

Items	Trial period (d)	Control	Treatment^1)^	SEM	p-value
Body weight (kg)	15	52.21	55.19	1.022	0.019
	30	64.77	69.13	1.813	0.043
Body length (cm)	15	64.33	64.87	1.203	0.665
	30	71.77	72.53	1.245	0.505
Withers height (cm)	15	79.95	79.85	0.852	0.901
	30	82.49	84.71	0.780	0.020
Heart girth (cm)	15	83.07	85.63	0.989	0.032
	30	90.06	93.04	1.541	0.088
Cannon circumference (cm)	15	11.59	11.90	0.266	0.292
	30	11.89	12.19	0.284	0.307
Hip width (cm)	15	11.89	13.51	0.338	0.001
	30	13.32	14.63	0.364	0.007
Starter intake (g/d)	1–15	40.05	43.63	2.835	0.242
	16–30	82.35	95.06	5.270	0.042
	1–30	61.20	69.35	3.690	0.058
ADG (kg/d)	1–15	0.71	0.89	0.289	0.140
	16–30	0.83	0.93	0.145	0.334
	1–30	0.77	0.91	0.057	0.036
Feed efficiency^2)^	1–15	0.61	0.77	0.255	0.100
	16–30	0.59	0.65	0.133	0.367
	1–30	0.60	0.70	0.075	0.040

1) The treatment group, calves received conventional diet and additives from co-fermented with edible plants and probiotics (30 g per head per day).

2) Average daily gain / (milk solids and starter intake). All calves were fed in individual buckets three times daily (135 g/L, 0830, 1430 and 2030) from 1 to 30 days of trial period (2.5 L/time from 1 to 7 days of trial period; 3 L/time from 8 to 21 days of trial period; 3.5 L/time from 22 to 30 days of trial period).

SEM, standard error of the mean; ADG, average daily gain.

**Table 4:** Summary of mean morbidity^1)^ rate due to bovine respiratory disease and calf diarrhea in Holstein calves

Items	Control	Treatment^2)^	SEM	p-value		

				Tri^3)^	Time	Tri×Time
Disease incidence (%)						
CD	80.00	20.00	-	-	-	-
BRD	30.00	10.00	-	-	-	-
Antibiotic, administrations/calf^4)^	1.80	0.50	0.562	0.040	-	-
Mean fecal score^5)^	1.20	0.70	0.145	0.022	0.008	0.250

1) Mean morbidity (%) = Percentage of calves with BRD or CD / Number of calves reared.

2) The treatment group, calves received conventional diet and additives from co-fermented with edible plants and probiotics (30g per head per day).

3) The treatment effect between the control group and the treatment group.

4) Antibiotic, administrations/calf = average number of times calves received antibiotics during the trial period.

5) Daily measurements of every ten-day fecal scores were condensed into means values.

SEM, standard error of the mean; CD, calf diarrhea; BRD, bovine respiratory disease.

**Table 5:** Effect of additives from co-fermented with edible plants and probiotics on plasma metabolites in Holstein calves

Items	Control	Treatment^1)^	SEM	p-value		

				Tri^2)^	Time	Tri×Time
Biochemical levels						
GLU (mmol/L)	5.36	5.75	0.102	0.013	0.070	0.473
TP (g/L)	60.71	62.99	0.825	0.045	0.010	0.924
ALB (g/L)	11.85	11.70	0.345	0.786	0.011	0.591
GLB (g/L)	49.05	51.10	0.771	0.022	0.027	0.957
ALT (IU/L)	7.65	7.61	0.191	0.843	0.876	0.296
AST (IU/L)	52.25	53.02	1.541	0.713	0.395	0.404
ALP (IU/L)	87.81	87.64	3.047	0.968	0.769	0.890
LDH (IU/L)	730.78	738.60	13.151	0.678	0.891	0.933
CK (IU/L)	131.82	132.00	3.354	0.969	0.401	0.602
Antioxidant levels						
T-AOC (U/mL)	3.27	4.09	0.107	<0.001	0.653	0.380
CAT (U/mL)	3.78	3.65	0.107	0.385	0.472	0.132
GSH-Px (U/mL)	168.85	189.56	1.193	<0.001	0.058	0.273
MDA (nmol/mL)	3.47	2.48	0.264	<0.001	0.276	0.884
T-SOD (U/mL)	163.70	174.78	2.192	0.002	0.858	0.517
Immunological levels						
IgA (g/L)	1.13	1.42	0.020	<0.001	<0.001	0.006
IgM (g/L)	2.17	2.19	0.045	0.693	<0.001	0.763
IgG (g/L)	10.71	11.88	0.233	0.001	<0.001	0.050
TNF-α (pg/mL)	240.54	225.24	5.179	0.047	0.390	0.272
IL-1β (pg/mL)	48.04	44.10	1.238	0.033	0.009	0.222
IL-6 (pg/mL)	44.44	41.38	0.360	<0.001	0.285	0.143
IFN-γ (pg/mL)	34.48	35.68	0.844	0.328	0.718	0.402
IL-10 (pg/mL)	31.33	33.86	0.893	0.021	0.067	0.233
HP (μg/mL)	7.57	7.34	0.226	0.490	0.433	0.990
SAA (μg/mL)	21.23	20.25	1.046	0.519	0.941	0.996

1) The treatment group, calves received conventional diet and additives from co-fermented with edible plants and probiotics (30 g per head per day).

2) The treatment effect between the control group and the treatment group.

SEM, standard error of the mean; GLU, glucose; TP, total protein; ALB, albumin; GLB, globulin; ALT, alanine transaminase; AST, aspartate transaminase; ALP, alkaline phosphatase; LDH, lactate dehydrogenase; CK, Creatine Kinase; T-AOC, total antioxidant capacity; CAT, catalase; GSH-Px, glutathione peroxidase; MDA, malondialdehyde; T-SOD, total superoxide dismutase; IgA, immunoglobulin A; IgM, immunoglobulin M; IgG, immunoglobulin G; TNF-α, tumor necrosis factor-α; IL-1β, interleukin-1β; IL-6, interleukin-6; IFN-γ, interferon-γ; IL-10, interleukin-10; HP, haptoglobin; SAA, serum amyloid A.

**Table 6:** Effect of additives from co-fermented with edible plants and probiotics on fecal fermentation parameters in Holstein calves

Items	Control	Treatment^1)^	SEM	p-value		

				Tri^2)^	Time	Tri×Time
pH	7.49	7.24	0.031	<0.001	<0.001	0.003
Total VFA (mmol/g of feces)	0.09	0.11	0.002	<0.001	<0.001	0.575
VFA (% of total VFA)						
Acetate	57.22	56.65	0.331	0.208	0.238	0.721
Propionate	27.19	26.83	0.233	0.287	<0.001	0.929
Butyrate	9.60	10.55	0.161	<0.001	<0.001	0.069
Isobutyrate	1.42	1.36	0.040	0.269	<0.001	0.944
Valerate	2.68	2.75	0.096	0.635	<0.001	0.625
Isovalerate	1.88	1.87	0.101	0.955	0.001	0.997

1) The treatment group, calves received conventional diet and additives from co-fermented with edible plants and probiotics (30 g per head per day).

2) The treatment effect between the control group and the treatment group.

SEM, standard error of the mean; VFA, volatile fatty acids.

## Data Availability

Upon reasonable request, the datasets of this study can be available from the corresponding author.

## SUPPLEMENTARY MATERIAL

**Supplement 1**. Summary of sequence data generated from rumen samples of 6 Control group and 6 Treatment calves.

**Supplement 2**. The relative abundance (%) of rumen microbial domain in calves.

**Supplement 3**. Changes in richness and diversity of bacteria in the rumen of calves.

**Supplement 4**. The relative abundance (%) of 10 most predominant phylum and genera in the rumen of calves.

**Supplement 5**. Significant differences of in rumen bacterial phyla and genera of calves.

**Supplement 6**. Significant correlation between rumen microbial and host phenotypic indicators.

**Supplement 7**. Significant differences in rumen KEGG pathway level 3 in calves.

**Supplement 8**. Significant differences in rumen KEGG modules in calves.

**Supplement 9**. The relative abundance (%) of rumen class-level CAZymes in calves.

**Supplement 10**. The significant differences of in rumen family-level CAZymes in calves.

**Supplement 11**. KEGG pathway enrichment analysis identified the significantly different metabolites between the Control group and the Treatment group.

**Supplement 12**. Identification of significantly different rumen metabolites of calves.

**Supplement 13**. Top 15 rumen metabolites identified by Random Forest Analysis.

**Supplement 14**. Significant correlation between rumen metabolites and host phenotypic indicators.

**Supplement 15**. Significant correlations between rumen bacteria and metabolites associated with host phenotypic indicators.

**Supplement 16**. Changes in richness and diversity of bacteria in the feces of Holstein calves.

**Supplement 17**. The relative abundance (%) of 10 most predominant phylum and genera in the feces of calves.

**Supplement 18**. Significant correlations between fecal bacteria and host phenotypic indicators.
