# Supplementary material for: The additive from co-fermented edible plants and probiotics improved calves’ growth performance and health by regulating antioxidant and gastrointestinal-microbiota
Source: Anim Biosci. 2025 Nov 14;39(5):250112. doi: 10.5713/ab.250112 (PMC13175069; doi:10.5713/ab.250112)
Supplement: Supplementary file 1 [file ab-250112-Supplement-1.pdf]

Supplement 1. Summary of sequence data generated from rumen samples of 6 Control group and 6 Treatment[1)] calves

Samples	Raw reads	Optimized reads	Contigs	N50(bp)
Control_1	44607666	42186208	258389	1584
Control_2	42699586	40693414	243680	1600
Control_3	42927010	40167314	244654	1558
Control_4	40407672	37738520	236858	1623
Control_5	44331950	41823884	261726	1693
Control_6	39686900	37607996	235393	1711
Treatment _1	39936220	37947290	250292	1556
Treatment _2	44290236	42278968	269578	1608
Treatment _3	44541284	42315314	269676	1618
Treatment _4	39415564	37300930	237415	1555
Treatment _5	44646064	42948684	272161	1612
Treatment _6	39538990	37459988	234575	1484
Total	507029142	480468510	3014397	19202
Mean	42252429	40039043	251200	1600
SEM	2264509	2268999	14470	61

[1)] The treatment group, calves received conventional diet and additives from co-fermented with edible plants and probiotics (30g per head per day).

SEM: standard error of the mean.
