# Supplementary material for: The additive from co-fermented edible plants and probiotics improved calves’ growth performance and health by regulating antioxidant and gastrointestinal-microbiota
Source: Anim Biosci. 2025 Nov 14;39(5):250112. doi: 10.5713/ab.250112 (PMC13175069; doi:10.5713/ab.250112)
Supplement: Supplementary file 2 [file ab-250112-Supplement-2.pdf]

**Supplement 2.** The relative abundance (%) of rumen microbial domain in calves

| Items | Control | Treatment[1)] |
|---|---|---|
| Bacteria | 90.80±3.662 | 93.13±1.945 |
| Viruses | 6.29±1.863 | 4.97±0.955 |
| Archaea | 2.89±1.810 | 1.89±1.023 |
| Eukaryota | 0.01±0.001 | 0.01±0.000 |

[1)] The treatment group, calves received conventional diet and additives from co-fermented with edible plants and probiotics (30g per head per day).
