# Supplementary material for: The additive from co-fermented edible plants and probiotics improved calves’ growth performance and health by regulating antioxidant and gastrointestinal-microbiota
Source: Anim Biosci. 2025 Nov 14;39(5):250112. doi: 10.5713/ab.250112 (PMC13175069; doi:10.5713/ab.250112)
Supplement: Supplementary file 3 [file ab-250112-Supplement-3.pdf]

**Supplement 3.** Changes in richness and diversity of bacteria in the rumen of calves

| Items | Control | Treatment[1)] | SEM | *P*-value |
|---|---|---|---|---|
| Ace | 12511.33 | 12278.17 | 87.555 | 0.089 |
| Chao1 | 12511.33 | 12278.17 | 87.555 | 0.089 |
| Shannon | 5.23 | 5.15 | 0.026 | 0.043 |
| Simpson | 0.03 | 0.03 | 0.000 | 0.001 |

[1)] The treatment group, calves received conventional diet and additives from co-fermented with edible plants and probiotics (30g per head per day).
