# Supplementary material for: The additive from co-fermented edible plants and probiotics improved calves’ growth performance and health by regulating antioxidant and gastrointestinal-microbiota
Source: Anim Biosci. 2025 Nov 14;39(5):250112. doi: 10.5713/ab.250112 (PMC13175069; doi:10.5713/ab.250112)
Supplement: Supplementary file 4 [file ab-250112-Supplement-4.pdf]

Supplement 4. The relative abundance (%) of 10 most predominant phylum and genera in the rumen of calves

Items	Control	Treatment[1)]
Phylum		
Bacillota	38.25±0.606	40.47±0.846
Bacteroidota	37.59±1.817	39.68±1.372
Uroviricota	5.58±1.645	4.39±0.847
Actinomycetota	2.6±0.613	2.76±0.376
Euryarchaeota	2.54±1.815	1.6±1.023
Thermodesulfobacteriota	1.95±0.547	2.01±0.323
Spirochaetota	1.69±0.059	1.51±0.063
Pseudomonadota	1.56±0.473	1.29±0.27
Fibrobacterota	1.43±0.195	1.25±0.232
Candidatus_Saccharibacteria	1.16±0.204	1.29±0.128
Others	5.64±0.726	3.75±0.1
Genus		
Prevotella	15.48±0.866	17.29±0.471
Candidatus_Cryptobacteroides	6.8±1.749	6.54±0.874
Ruminococcus	4.81±0.156	5.37±0.292
unclassified_c__Caudoviricetes	5.36±1.513	4.24±0.776
Selenomonas	2.98±1.028	2.75±0.554
Xylanibacter	2.18±0.244	2.69±0.091
Bacteroides	2.34±0.110	2.46±0.068
Eubacterium	1.9±0.164	2.1±0.085
Succiniclasticum	1.84±0.523	1.89±0.318
Butyrivibrio	1.89±0.794	1.83±0.362
Others	54.41±0.798	52.86±0.981

[1)] The treatment group, calves received conventional diet and additives from co-fermented with edible plants and probiotics (30g per head per day).
