# Supplementary material for: The additive from co-fermented edible plants and probiotics improved calves’ growth performance and health by regulating antioxidant and gastrointestinal-microbiota
Source: Anim Biosci. 2025 Nov 14;39(5):250112. doi: 10.5713/ab.250112 (PMC13175069; doi:10.5713/ab.250112)
Supplement: Supplementary file 5 [file ab-250112-Supplement-5.pdf]

Supplement 5. Significant differences of in rumen bacterial phyla and genera of calves

Items	Control	Treatment[1)]	LDA-value[2)]	*P*-value
Differences in bacterial phylum level				
Bacteroidota	37.59±1.817	39.68±1.372	3.77	0.037
Synergistota	1.33±0.354	0.89±0.125	3.46	0.004
Kiritimatiellota	0.67±0.286	0.37±0.079	3.30	0.016
Lentisphaerota	0.57±0.225	0.29±0.057	3.26	0.004
Spirochaetota	1.69±0.059	1.51±0.063	3.08	0.004
Verrucomicrobiota	0.34±0.104	0.17±0.029	3.02	0.004
Planctomycetota	0.25±0.082	0.13±0.021	2.88	0.004
Thermosulfidibacterota	0.01±0.000	0.00±0.000	2.35	0.010
Candidatus_Latescibacterota	0.06±0.025	0.03±0.007	2.28	0.006
Candidatus_Hydrogenedentota	0.07±0.025	0.04±0.008	2.27	0.010
Candidatus_Caldatribacteriota	0.01±0.001	0.00±0.000	2.26	0.020
Abditibacteriota	0.01±0.000	0.00±0.000	2.06	0.004
Elusimicrobiota	0.07±0.008	0.05±0.01	2.02	0.016
Candidatus_Adlerbacteria	0.00±0.000	0.01±0.000	2.02	0.025
Candidatus_Paceibacterota	0.04±0.005	0.02±0.001	2.01	0.004
Candidatus_Omnitrophota	0.04±0.011	0.02±0.002	2.00	0.004
Differences in bacterial genus level				
Prevotella	15.48±0.866	17.29±0.471	3.84	0.025
Bacillus	0.07±0.015	0.62±0.116	3.47	0.004
Ruminococcus	4.81±0.156	5.37±0.292	3.37	0.006
Xylanibacter	2.18±0.244	2.69±0.091	3.36	0.006
Candidatus_Colimorpha	1.61±0.326	1.30±0.136	3.27	0.006
Cloacibacillus	0.70±0.195	0.38±0.056	3.26	0.004
Candidatus_Colisoma	0.63±0.265	0.35±0.074	3.19	0.016
Victivallis	0.41±0.158	0.20±0.039	3.07	0.004
Candidatus_Limimorpha	1.09±0.095	1.32±0.096	3.05	0.004

Hallella	0.23±0.025	0.43±0.043	3.01	0.004
Angelakisella	0.43±0.141	0.30±0.047	2.84	0.025
Candidatus_Weimeria	0.14±0.053	0.26±0.015	2.84	0.006
Schwartzia	0.48±0.13	0.37±0.055	2.80	0.037
Paraprevotella	0.24±0.04	0.35±0.018	2.76	0.004
Galactobacillus	0.14±0.017	0.25±0.021	2.76	0.004
Porcincola	0.26±0.031	0.35±0.033	2.66	0.004
Candidatus_Colicola	0.23±0.065	0.15±0.017	2.66	0.004
Synergistes	0.19±0.049	0.11±0.015	2.64	0.004
Candidatus_Merdousia	0.12±0.046	0.04±0.008	2.63	0.004
Lachnoclostridium	0.41±0.035	0.50±0.039	2.61	0.004
Sarcina	0.23±0.03	0.31±0.035	2.58	0.010
Hornefia	0.10±0.01	0.16±0.016	2.49	0.004
Oribacterium	0.36±0.042	0.43±0.018	2.49	0.037
Candidatus_Egerieousia	0.16±0.008	0.11±0.008	2.48	0.004
Blautia	0.79±0.026	0.85±0.041	2.40	0.010
Candidatus_Spyradenecus	0.10±0.043	0.06±0.012	2.37	0.016
Atopobium	0.09±0.028	0.13±0.022	2.29	0.025
Megasphaera	0.17±0.034	0.21±0.027	2.28	0.025
Bilifractor	0.08±0.003	0.12±0.008	2.28	0.004
Candidatus_Limimonas	0.11±0.03	0.07±0.012	2.24	0.010
Stecheria	0.12±0.008	0.16±0.012	2.23	0.004
unclassified_p__Candidatus_Latescibacterota	0.06±0.025	0.03±0.007	2.19	0.006
Solobacterium	0.54±0.031	0.58±0.016	2.18	0.004
unclassified_p__Candidatus_Hydrogenedentota	0.06±0.024	0.04±0.008	2.18	0.010
Oligosphaera	0.06±0.023	0.03±0.007	2.17	0.004
Leyella	0.08±0.006	0.11±0.008	2.16	0.004
Phascolarctobacterium	0.39±0.018	0.42±0.022	2.14	0.037
Akkermansia	0.08±0.008	0.06±0.009	2.13	0.004
Candidatus_Caccocola	0.05±0.014	0.02±0.004	2.08	0.004

Rhodopirellula	0.04±0.015	0.02±0.004	2.06	0.004
Anaerolactibacter	0.08±0.007	0.1±0.006	2.05	0.004
Oliverpabstia	0.02±0.005	0.04±0.006	2.03	0.004
Catonella	0.04±0.007	0.06±0.005	2.02	0.006

[1)] The treatment group, calves received conventional diet and additives from co-fermented with edible plants and probiotics (30g per head per day).

[2)] Linear discriminant analysis > 2 and $P < 0.05$ are considered significantly different.
