# Supplementary material for: The additive from co-fermented edible plants and probiotics improved calves’ growth performance and health by regulating antioxidant and gastrointestinal-microbiota
Source: Anim Biosci. 2025 Nov 14;39(5):250112. doi: 10.5713/ab.250112 (PMC13175069; doi:10.5713/ab.250112)
Supplement: Supplementary file 6 [file ab-250112-Supplement-6.pdf]

Supplement 6. Significant correlation between rumen microbial and host phenotypic indicators[1)]

Items	Indicator	Correlation coefficient	*P*-value
g__*Ruminococcus*	ADG	0.97	0.001
g__*Lachnoclostridium*	ADG	0.97	0.001
g__*Galactobacillus*	ADG	0.88	0.020
g__*Porcincola*	ADG	0.88	0.020
g__*Xylanibacter*	IL-6	-0.94	0.017

[1)] Significant correlation conditions: $|R| > 0.5$, $P < 0.05$.
