# Supplementary material for: The additive from co-fermented edible plants and probiotics improved calves’ growth performance and health by regulating antioxidant and gastrointestinal-microbiota
Source: Anim Biosci. 2025 Nov 14;39(5):250112. doi: 10.5713/ab.250112 (PMC13175069; doi:10.5713/ab.250112)
Supplement: Supplementary file 7 [file ab-250112-Supplement-7.pdf]

Supplement 7. Significant differences in rumen KEGG pathway level 3 in calves

Items	Control	Treatment[1)]	LDA-value[2)]	*P*-value
Two-component system	2.01±0.022	2.09±0.021	2.66	0.004
Starch and sucrose metabolism	1.04±0.021	1.12±0.019	2.60	0.004
Pyrimidine metabolism	1.38±0.082	1.29±0.039	2.59	0.037
Biosynthesis of cofactors	3.13±0.016	3.09±0.02	2.39	0.004
Phosphotransferase system (PTS)	0.2±0.006	0.22±0.012	2.18	0.004
Lipopolysaccharide biosynthesis	0.39±0.012	0.36±0.008	2.14	0.004
Apoptosis	0.09±0.005	0.11±0.01	2.03	0.004
Folate biosynthesis	0.46±0.007	0.44±0.005	2.03	0.004

[1)] The treatment group, calves received conventional diet and additives from co-fermented with edible plants and probiotics (30g per head per day).

[2)] Linear discriminant analysis > 2 and $P < 0.05$ are considered significantly different.
