# Supplementary material for: The additive from co-fermented edible plants and probiotics improved calves’ growth performance and health by regulating antioxidant and gastrointestinal-microbiota
Source: Anim Biosci. 2025 Nov 14;39(5):250112. doi: 10.5713/ab.250112 (PMC13175069; doi:10.5713/ab.250112)
Supplement: Supplementary file 8 [file ab-250112-Supplement-8.pdf]

Supplement 8. Significant differences in rumen KEGG modules in calves

Items	Control	Treatment[1)]	LDA-value[2)]	*P*-value
Incomplete reductive citrate cycle, acetyl-CoA => oxoglutarate (M00620)	0.93±0.011	0.89±0.018	2.27	0.025
UDP-N-acetyl-D-glucosamine biosynthesis, prokaryotes, glucose => UDP-GlcNAc (M00909)	0.89±0.014	0.92±0.013	2.15	0.016
Trehalose biosynthesis, D-glucose 1P => trehalose (M00565)	0.59±0.010	0.64±0.015	2.36	0.004
Valine/isoleucine biosynthesis, pyruvate => valine / 2-oxobutanoate => isoleucine (M00019)	0.67±0.009	0.70±0.006	2.20	0.004
Vancomycin resistance, D-Ala-D-Lac type (M00651)	0.44±0.010	0.46±0.010	2.08	0.016
ADP-L-glycero-D-manno-heptose biosynthesis (M00064)	0.18±0.004	0.16±0.007	2.05	0.004
Glycogen degradation, glycogen => glucose-6P (M00855)	0.7±0.018	0.79±0.022	2.68	0.004
Isoleucine biosynthesis, threonine => 2-oxobutanoate => isoleucine (M00570)	0.73±0.011	0.77±0.007	2.23	0.004
Tryptophan biosynthesis, chorismate => tryptophan (M00023)	0.72±0.015	0.76±0.011	2.36	0.004
Biotin biosynthesis, pimeloyl-ACP/CoA => biotin (M00123)	0.28±0.019	0.26±0.008	2.07	0.037
Biotin biosynthesis, BioW pathway, pimelate => pimeloyl-CoA => biotin (M00577)	0.28±0.018	0.27±0.008	2.06	0.037
Tetracycline resistance, efflux pump Tet38 (M00704)	0.06±0.013	0.08±0.008	2.02	0.010
Pyruvate oxidation, pyruvate => acetyl-CoA (M00307)	0.5±0.017	0.48±0.009	2.02	0.037
Cobalamin biosynthesis, aerobic, uroporphyrinogen III => precorrin 2 => cobyrinate a,c-diamide (M00925)	0.49±0.016	0.45±0.009	2.18	0.004
F-type ATPase, prokaryotes and chloroplasts (M00157)	1.26±0.024	1.32±0.031	2.41	0.016
Glycogen biosynthesis, glucose-1P => glycogen/starch (M00854)	0.84±0.018	0.88±0.013	2.33	0.004
Methanogenesis, methylamine/dimethylamine/trimethylamine => methane (M00563)	0.39±0.019	0.36±0.010	2.18	0.006
Isoleucine biosynthesis, pyruvate => 2-oxobutanoate (M00535)	0.35±0.006	0.37±0.003	2.03	0.004
Leucine biosynthesis, 2-oxoisovalerate => 2-oxoisocaproate (M00432)	0.42±0.010	0.44±0.007	2.03	0.010

[1] The treatment group, calves received conventional diet and additives from co-fermented with edible plants and probiotics (30g per head per day).

[2] Linear discriminant analysis > 2 and $P < 0.05$ are considered significantly different.
