# Supplementary material for: The additive from co-fermented edible plants and probiotics improved calves’ growth performance and health by regulating antioxidant and gastrointestinal-microbiota
Source: Anim Biosci. 2025 Nov 14;39(5):250112. doi: 10.5713/ab.250112 (PMC13175069; doi:10.5713/ab.250112)
Supplement: Supplementary file 9 [file ab-250112-Supplement-9.pdf]

Supplement 9. The relative abundance (%) of rumen class-level CAZymes in calves

Items	Control	Treatment[1)]
Glycoside Hydrolases	46.93±3.162	47.75±1.900
Glycosyl Transferases	30.84±4.927	29.82±2.963
Carbohydrate Esterases	14.80±1.229	15.00±0.782
Auxiliary Activities	3.20±0.164	3.18±0.147
Carbohydrate-Binding Modules	2.90±0.248	2.97±0.118
Polysaccharide Lyases	1.28±0.147	1.24±0.079
Cellulosome Modules	0.05±0.019	0.05±0.013

[1)] The treatment group, calves received conventional diet and additives from co-fermented with edible plants and probiotics (30g per head per day).
