# Supplementary material for: The additive from co-fermented edible plants and probiotics improved calves’ growth performance and health by regulating antioxidant and gastrointestinal-microbiota
Source: Anim Biosci. 2025 Nov 14;39(5):250112. doi: 10.5713/ab.250112 (PMC13175069; doi:10.5713/ab.250112)
Supplement: Supplementary file 10 [file ab-250112-Supplement-10.pdf]

Supplement 10. The significant differences of in rumen family-level CAZymes in calves

Items	Control	Treatment[1)]	LDA-value[2)]	*P*-value
Glycoside Hydrolases, GHs				
GH1	0.53±0.061	0.66±0.086	2.84	0.025
GH77	0.82±0.073	0.95±0.051	2.80	0.010
GH163	0.31±0.083	0.18±0.020	2.79	0.004
GH32	0.59±0.051	0.69±0.043	2.69	0.016
GH39	0.33±0.067	0.25±0.030	2.64	0.016
GH13_20	0.27±0.050	0.33±0.024	2.53	0.025
GH13_9	0.34±0.015	0.39±0.024	2.49	0.004
GH13_14	0.20±0.009	0.26±0.018	2.46	0.004
GH5_4	0.30±0.015	0.35±0.018	2.44	0.004
GH115	0.38±0.032	0.44±0.025	2.43	0.010
GH76	0.18±0.020	0.23±0.015	2.42	0.004
GH129	0.13±0.035	0.09±0.009	2.37	0.004
GH108	0.23±0.025	0.19±0.022	2.34	0.025
GH35	0.25±0.019	0.29±0.014	2.33	0.004
GH165	0.07±0.016	0.03±0.008	2.29	0.004
GH148	0.10±0.026	0.06±0.01	2.29	0.004
GH13_31	0.27±0.024	0.31±0.018	2.26	0.025
GH13_5	0.08±0.014	0.12±0.016	2.22	0.006
GH43_3	0.08±0.009	0.11±0.014	2.20	0.006
GH13_38	0.25±0.024	0.28±0.019	2.17	0.016
GH74	0.12±0.012	0.15±0.010	2.14	0.006
GH167	0.06±0.018	0.03±0.007	2.13	0.010
GH13_28	0.10±0.006	0.13±0.014	2.10	0.016
GH13_11	0.12±0.010	0.14±0.012	2.08	0.010
GH31_15	0.16±0.004	0.18±0.009	2.05	0.004
GH8	0.16±0.010	0.18±0.011	2.04	0.010

GH5_36	0.05±0.014	0.02±0.007	2.04	0.025
GH31_1	0.08±0.007	0.10±0.009	2.03	0.010
GH30_4	0.07±0.008	0.09±0.008	2.03	0.010
GH13_4	0.14±0.009	0.16±0.015	2.02	0.037
GH13_6	0.10±0.013	0.12±0.008	2.02	0.016
GH43_31	0.05±0.006	0.07±0.007	2.01	0.004
Glycosyl Transferases, GTs				
GT35	0.87±0.018	0.97±0.034	2.73	0.004
GT5	1.26±0.016	1.31±0.021	2.36	0.010
GT51	0.53±0.023	0.58±0.013	2.35	0.004
GT3	0.15±0.011	0.18±0.011	2.19	0.004
Carbohydrate-Binding Modules, CBMs				
CBM16	0.09±0.024	0.06±0.006	2.21	0.004
CBM13	0.10±0.010	0.12±0.012	2.13	0.006
Carbohydrate Esterases, CEs				
CE14	0.27±0.040	0.22±0.019	2.39	0.037
CE2	0.49±0.027	0.54±0.033	2.31	0.037

[1)] The treatment group, calves received conventional diet and additives from co-fermented with edible plants and probiotics (30g per head per day).

[2)] Linear discriminant analysis > 2 and $P < 0.05$ are considered significantly different.
