# Supplementary material for: The additive from co-fermented edible plants and probiotics improved calves’ growth performance and health by regulating antioxidant and gastrointestinal-microbiota
Source: Anim Biosci. 2025 Nov 14;39(5):250112. doi: 10.5713/ab.250112 (PMC13175069; doi:10.5713/ab.250112)
Supplement: Supplementary file 11 [file ab-250112-Supplement-11.pdf]

Supplement 11. KEGG pathway enrichment analysis identified the significantly different metabolites between the Control group and the Treatment[1)] group

ID	Description	Ratio in study[2)]	Ratio in pop[3)]	Enrich factor[4)]	*P*-value
ko00380	Tryptophan metabolism	5.74%	1.81%	0.08	0.035
ko00750	Vitamin B6 metabolism	3.28%	0.63%	0.14	0.034
ko00740	Riboflavin metabolism	3.28%	0.52%	0.17	0.021

1) The treatment group, calves received conventional diet and additives from co-fermented with edible plants and probiotics (30g per head per day).

2) Ratio in study = the number of differential metabolites associated with this pathway/the number of differential metabolites annotated by KEGG.

3) Ratio in pop = the ratio of the number of all metabolites associated with this pathway to the number of all metabolites annotated by KEGG.

4) Enrich factor = Metabolite ratio/pop ratio.
