# Supplementary material for: The additive from co-fermented edible plants and probiotics improved calves’ growth performance and health by regulating antioxidant and gastrointestinal-microbiota
Source: Anim Biosci. 2025 Nov 14;39(5):250112. doi: 10.5713/ab.250112 (PMC13175069; doi:10.5713/ab.250112)
Supplement: Supplementary file 12 [file ab-250112-Supplement-12.pdf]

Supplement 12. Identification of significantly different rumen metabolites of calves

KEGG pathway ID	KEGG Compound ID	Metabolite	FC[1)]	VIP	*P*-value	Regulation
ko00380	C00954	Indoleacetic acid	1.02	1.21	0.001	up
	C00954	Indole-3-acetic acid	1.09	2.56	<0.001	up
	C00328	L-Kynurenine	0.98	1.04	0.002	down
	C10164	Picolinic acid	1.03	1.36	<0.001	up
	C05655	5-(2'-Carboxyethyl)-4,6-Dihydroxypicolinate	0.98	1.07	0.001	down
	C05637	Quinoline-4,8-diol	1.05	2.26	<0.001	up
	C05653	2-formamidobenzoic acid	1.02	1.24	<0.001	up
	C00078	L-Tryptophan	0.99	1.03	<0.001	down
ko00750	C04604	3-Hydroxy-2-methylpyridine-4,5-dicarboxylate	0.98	1.12	0.008	down
	C06055	O-Phospho-4-hydroxy-L-threonine	1.02	1.12	<0.001	up
	C06051	Isopyridoxal	1.02	1.09	<0.001	up
	C00314	Pyridoxine	1.22	3.94	<0.001	up
ko00740	C01727	Lumichrome	1.09	2.65	<0.001	up
	C00255	Riboflavin	0.97	1.45	0.001	down
	C03114	5,6-dimethylbenzimidazole	1.02	1.27	<0.001	up
	C00061	Flavin mononucleotide	1.02	1.04	0.031	up

[1)] Ratio of the Treatment group to the Control group.

FC, Fold Change; VIP, Variable Importance in Projection.
