# Supplementary material for: The additive from co-fermented edible plants and probiotics improved calves’ growth performance and health by regulating antioxidant and gastrointestinal-microbiota
Source: Anim Biosci. 2025 Nov 14;39(5):250112. doi: 10.5713/ab.250112 (PMC13175069; doi:10.5713/ab.250112)
Supplement: Supplementary file 13 [file ab-250112-Supplement-13.pdf]

Supplement 13. Top 15 rumen metabolites identified by Random Forest Analysis

KEGG Compound ID	Metabolite	FC[1)]	VIP	MDA
C05637	Quinoline-4,8-diol	1.05	2.26	0.016
C04294	4-methyl-5-thiazoleethanol	1.21	4.09	0.012
C05954	Prostaglandin b2	0.97	1.52	0.012
C00517	Palmitaldehyde	0.96	1.67	0.012
C01279	4-amino-5-hydroxymethyl-2-methylpyrimidine	0.93	2.33	0.012
C11355	4-amino-4-deoxychorismate	1.06	1.91	0.011
C00328	L-Kynurenine	0.98	1.04	0.011
C00255	Riboflavin	0.97	1.45	0.011
C11457	Dihydro-3-coumaric acid	1.03	1.72	0.011
C00993	Ala-Ala	1.08	2.10	0.011
C05598	Phenaceturic acid	1.08	2.26	0.010
C00163	Propionic Acid	1.05	1.66	0.010
C00120	Vitamin b7	1.11	2.91	0.010
C01727	Lumichrome	1.09	2.65	0.010
C00954	Indole-3-acetic acid	1.09	2.56	0.010

[1)] Ratio of the Treatment group to the Control group.

FC, Fold Change; VIP, Variable Importance in Projection; MDA, Mean Decrease Accuracy.
