# Supplementary material for: The additive from co-fermented edible plants and probiotics improved calves’ growth performance and health by regulating antioxidant and gastrointestinal-microbiota
Source: Anim Biosci. 2025 Nov 14;39(5):250112. doi: 10.5713/ab.250112 (PMC13175069; doi:10.5713/ab.250112)
Supplement: Supplementary file 14 [file ab-250112-Supplement-14.pdf]

Supplement 14. Significant correlation between rumen metabolites and host phenotypic indicators[1)]

Indicator	Metabolite	Correlation coefficient	*P*-value
ADG	Indole-3-acetic acid	0.77	0.004
	L-Tryptophan	-0.76	0.004
	3-Hydroxy-2-methylpyridine-4,5-dicarboxylate	-0.76	0.004
	5-(2'-Carboxyethyl)-4,6-Dihydroxypicolinate	-0.74	0.006
	Indoleacetic acid	0.72	0.009
	O-Phospho-4-hydroxy-L-threonine	0.70	0.011
	2-formamidobenzoic acid	0.70	0.012
	Pyridoxine	0.64	0.024
	Lumichrome	0.60	0.037
	4-amino-4-deoxychorismate	0.59	0.042
	4-methyl-5-thiazoleethanol	0.58	0.050
Feed efficiency	Prostaglandin b2	-0.91	<0.001
	Phenaceturic acid	0.87	<0.001
	Quinoline-4,8-diol	0.87	<0.001
	Ala-Ala	0.85	0.001
	Propionic Acid	0.83	0.002
	Isopyridoxal	0.83	0.002
	Dihydro-3-coumaric acid	0.82	0.002
	4-amino-5-hydroxymethyl-2-methylpyrimidine	-0.82	0.002
	Palmitaldehyde	-0.81	0.002
	Pyridoxine	0.81	0.002
	Vitamin b7	0.80	0.003
	4-methyl-5-thiazoleethanol	0.78	0.004
	Picolinic acid	0.78	0.005

	Indole-3-acetic acid	0.76	0.007
	5-(2'-Carboxyethyl)-4,6-Dihydroxypicolinate	-0.74	0.008
	4-amino-4-deoxychorismate	0.73	0.010
	O-Phospho-4-hydroxy-L-threonine	0.73	0.010
	L-Tryptophan	-0.70	0.015
	Indoleacetic acid	0.68	0.019
	Lumichrome	0.67	0.020
	2-formamidobenzoic acid	0.67	0.020
GLB	5-(2'-Carboxyethyl)-4,6-Dihydroxypicolinate	-0.75	0.005
	O-Phospho-4-hydroxy-L-threonine	0.66	0.021
	Quinoline-4,8-diol	0.64	0.025
	Prostaglandin b2	-0.63	0.029
	2-formamidobenzoic acid	0.61	0.035
	4-methyl-5-thiazoleethanol	0.60	0.041
	L-Tryptophan	-0.59	0.044
GLU	4-amino-5-hydroxymethyl-2-methylpyrimidine	-0.83	0.001
	Phenaceturic acid	0.72	0.009
	Ala-Ala	0.70	0.012
	Pyridoxine	0.69	0.012
	Quinoline-4,8-diol	0.69	0.014
	Isopyridoxal	0.68	0.016
	Prostaglandin b2	-0.66	0.019
	Palmitaldehyde	-0.64	0.025
	4-methyl-5-thiazoleethanol	0.64	0.025
	Picolinic acid	0.63	0.029
	Dihydro-3-coumaric acid	0.62	0.030
	4-amino-4-deoxychorismate	0.61	0.035
	5-(2'-Carboxyethyl)-4,6-Dihydroxypicolinate	-0.58	0.046
	2-formamidobenzoic acid	0.58	0.049

GSH-pX	Ala-Ala	0.91	<0.001
	2-formamidobenzoic acid	0.91	<0.001
	Palmitaldehyde	-0.90	<0.001
	4-amino-4-deoxychorismate	0.90	<0.001
	Dihydro-3-coumaric acid	0.87	<0.001
	Propionic Acid	0.86	0.001
	Pyridoxine	0.85	0.001
	Isopyridoxal	0.85	0.001
	Quinoline-4,8-diol	0.83	0.001
	Picolinic acid	0.83	0.001
	Prostaglandin b2	-0.83	0.002
	L-Tryptophan	-0.82	0.002
	Vitamin b7	0.78	0.005
	Phenaceturic acid	0.77	0.005
	4-amino-5-hydroxymethyl-2-methylpyrimidine	-0.76	0.007
	O-Phospho-4-hydroxy-L-threonine	0.74	0.008
	Indole-3-acetic acid	0.71	0.013
	Indoleacetic acid	0.70	0.015
	5-(2'-Carboxyethyl)-4,6-Dihydroxypicolinate	-0.65	0.026
	4-methyl-5-thiazoleethanol	0.64	0.028
	Lumichrome	0.60	0.043
IgA	2-formamidobenzoic acid	0.92	<0.001
	Quinoline-4,8-diol	0.85	0.001
	Ala-Ala	0.85	0.001
	Prostaglandin b2	-0.84	0.001
	Propionic Acid	0.83	0.002
	O-Phospho-4-hydroxy-L-threonine	0.83	0.002
	4-amino-4-deoxychorismate	0.81	0.002
	Phenaceturic acid	0.80	0.003

	Dihydro-3-coumaric acid	0.78	0.004
	Isopyridoxal	0.78	0.005
	Picolinic acid	0.77	0.005
	L-Tryptophan	-0.76	0.006
	Palmitaldehyde	-0.76	0.007
	Vitamin b7	0.71	0.013
	5-(2'-Carboxyethyl)-4,6-Dihydroxypicolinate	-0.70	0.015
	4-amino-5-hydroxymethyl-2-methylpyrimidine	-0.69	0.017
	Lumichrome	0.66	0.022
	Pyridoxine	0.66	0.022
	4-methyl-5-thiazoleethanol	0.64	0.030
	Indoleacetic acid	0.64	0.030
IgG	Indoleacetic acid	0.81	0.002
	O-Phospho-4-hydroxy-L-threonine	0.71	0.013
	Isopyridoxal	0.68	0.019
	Palmitaldehyde	-0.66	0.022
	4-amino-5-hydroxymethyl-2-methylpyrimidine	-0.66	0.022
	Lumichrome	0.64	0.028
	Indole-3-acetic acid	0.64	0.030
	Ala-Ala	0.62	0.037
	Phenaceturic acid	0.59	0.049
	Vitamin b7	0.59	0.049
	Pyridoxine	0.59	0.049
T-AOC	Indoleacetic acid	0.89	<0.001
	Lumichrome	0.83	0.002
	O-Phospho-4-hydroxy-L-threonine	0.73	0.010
	3-Hydroxy-2-methylpyridine-4,5-dicarboxylate	-0.66	0.022

	4-amino-5-hydroxymethyl-2-methylpyrimidine	-0.64	0.030
	Isopyridoxal	0.62	0.037
TNF-α	Picolinic acid	-0.84	0.001
	L-Tryptophan	0.84	0.001
	Dihydro-3-coumaric acid	-0.83	0.002
	4-amino-4-deoxychorismate	-0.82	0.002
	Pyridoxine	-0.82	0.002
	Propionic Acid	-0.81	0.002
	5-(2'-Carboxyethyl)-4,6-Dihydroxypicolinate	0.80	0.003
	4-methyl-5-thiazoleethanol	-0.79	0.004
	2-formamidobenzoic acid	-0.73	0.009
	Prostaglandin b2	0.73	0.010
	Indole-3-acetic acid	-0.73	0.010
	Palmitaldehyde	0.71	0.013
	Quinoline-4,8-diol	-0.71	0.013
	Lumichrome	-0.71	0.013
	Vitamin b7	-0.69	0.016
	Phenaceturic acid	-0.68	0.019
	Ala-Ala	-0.67	0.020
	4-amino-5-hydroxymethyl-2-methylpyrimidine	0.65	0.026
	O-Phospho-4-hydroxy-L-threonine	-0.64	0.030
	Indoleacetic acid	-0.62	0.037
	Isopyridoxal	-0.62	0.037
T-SOD	4-amino-4-deoxychorismate	0.79	0.002
	Palmitaldehyde	-0.77	0.003
	Dihydro-3-coumaric acid	0.75	0.005
	Indoleacetic acid	0.74	0.006
	Isopyridoxal	0.72	0.009

2-formamidobenzoic acid	0.71	0.009
Ala-Ala	0.71	0.010
Picolinic acid	0.71	0.010
Propionic Acid	0.68	0.014
Pyridoxine	0.67	0.016
Lumichrome	0.66	0.020
Vitamin b7	0.65	0.021
4-amino-5-hydroxymethyl-2-methylpyrimidine	-0.64	0.026
Indole-3-acetic acid	0.61	0.036
L-Tryptophan	-0.59	0.045

[1)] Significant correlation = $|R| > 0.5$, $P < 0.05$.
