# Supplementary material for: The additive from co-fermented edible plants and probiotics improved calves’ growth performance and health by regulating antioxidant and gastrointestinal-microbiota
Source: Anim Biosci. 2025 Nov 14;39(5):250112. doi: 10.5713/ab.250112 (PMC13175069; doi:10.5713/ab.250112)
Supplement: Supplementary file 15 [file ab-250112-Supplement-15.pdf]

Supplement 15. Significant correlations between rumen bacteria and metabolites associated with host phenotypic indicators[1)]

Microorganism	Metabolite	Correlation coefficient	*P*-value
g__*Galactobacillus*	Picolinic acid	0.85	0.001
	Dihydro-3-coumaric acid	0.81	0.002
	5-(2'-Carboxyethyl)-4,6-Dihydroxypicolinate	-0.81	0.002
	Indole-3-acetic acid	0.80	0.003
	4-amino-5-hydroxymethyl-2-methylpyrimidine	-0.80	0.003
	2-formamidobenzoic acid	0.78	0.004
	Pyridoxine	0.78	0.004
	Quinoline-4,8-diol	0.77	0.005
	Propionic Acid	0.76	0.006
	Palmitaldehyde	-0.76	0.007
	Isopyridoxal	0.75	0.007
	Indoleacetic acid	0.73	0.009
	Ala-Ala	0.71	0.013
	Phenaceturic acid	0.69	0.016
	4-methyl-5-thiazoleethanol	0.65	0.026
	Lumichrome	0.64	0.030
	Vitamin b7	0.62	0.035
g__*Lachnoclostridium*	4-amino-4-deoxychorismate	0.83	0.002
	Picolinic acid	0.82	0.002
	Indole-3-acetic acid	0.79	0.004
	Dihydro-3-coumaric acid	0.76	0.006
	Quinoline-4,8-diol	0.74	0.008
	Palmitaldehyde	-0.74	0.008

	Pyridoxine	0.74	0.008
	4-amino-5-hydroxymethyl-2-methylpyrimidine	-0.73	0.009
	5-(2'-Carboxyethyl)-4,6-Dihydroxypicolinate	-0.71	0.012
	Isopyridoxal	0.71	0.013
	Propionic Acid	0.70	0.015
	2-formamidobenzoic acid	0.69	0.016
	Ala-Ala	0.66	0.022
	Indoleacetic acid	0.66	0.024
	Phenaceturic acid	0.62	0.037
g__*Porcincola*	4-amino-4-deoxychorismate	0.87	<0.001
	Picolinic acid	0.86	0.001
	Dihydro-3-coumaric acid	0.80	0.003
	Indole-3-acetic acid	0.80	0.003
	Pyridoxine	0.78	0.004
	4-amino-5-hydroxymethyl-2-methylpyrimidine	-0.78	0.005
	Quinoline-4,8-diol	0.76	0.006
	Palmitaldehyde	-0.76	0.006
	5-(2'-Carboxyethyl)-4,6-Dihydroxypicolinate	-0.76	0.007
	Propionic Acid	0.74	0.008
	2-formamidobenzoic acid	0.74	0.008
	Isopyridoxal	0.73	0.010
	Ala-Ala	0.70	0.015
	Indoleacetic acid	0.67	0.020
	Phenaceturic acid	0.66	0.024
	4-methyl-5-thiazoleethanol	0.62	0.037
g__*Ruminococcus*	Quinoline-4,8-diol	0.83	0.001

	Indole-3-acetic acid	0.73	0.009
	4-amino-4-deoxychorismate	0.73	0.010
	Picolinic acid	0.73	0.010
	5-(2'-Carboxyethyl)-4,6-Dihydroxypicolinate	-0.73	0.010
	2-formamidobenzoic acid	0.72	0.011
	Propionic Acid	0.69	0.017
	Dihydro-3-coumaric acid	0.67	0.020
	Phenaceturic acid	0.67	0.020
	Palmitaldehyde	-0.66	0.022
	Isopyridoxal	0.66	0.022
	Pyridoxine	0.66	0.024
	Ala-Ala	0.64	0.028
	4-amino-5-hydroxymethyl-2-methylpyrimidine	-0.64	0.030
	Indoleacetic acid	0.59	0.046
g__Xylanibacter	Vitamin b7	0.92	<0.001
	4-methyl-5-thiazoleethanol	0.85	0.001
	Phenaceturic acid	0.83	0.002
	Quinoline-4,8-diol	0.82	0.002
	Lumichrome	0.81	0.002
	Ala-Ala	0.80	0.003
	4-amino-5-hydroxymethyl-2-methylpyrimidine	-0.80	0.003
	Isopyridoxal	0.80	0.003
	Indole-3-acetic acid	0.79	0.004
	Indoleacetic acid	0.78	0.004
	Pyridoxine	0.76	0.006
	Palmitaldehyde	-0.75	0.007
	Propionic Acid	0.69	0.017

	5-(2'-Carboxyethyl)-4,6-Dihydroxypicolinate	-0.69	0.017
	2-formamidobenzoic acid	0.67	0.020
	Picolinic acid	0.64	0.028
	Dihydro-3-coumaric acid	0.64	0.030

[1)] Significant correlation = $|R| > 0.5$, $P < 0.05$.
