# Supplementary material for: The additive from co-fermented edible plants and probiotics improved calves’ growth performance and health by regulating antioxidant and gastrointestinal-microbiota
Source: Anim Biosci. 2025 Nov 14;39(5):250112. doi: 10.5713/ab.250112 (PMC13175069; doi:10.5713/ab.250112)
Supplement: Supplementary file 16 [file ab-250112-Supplement-16.pdf]

Supplement 16. Changes in richness and diversity of bacteria in the feces of Holstein calves

Items	Control	Treatment[1)]	SEM	*P*-value
Ace	494.08	729.72	36.519	0.007
Chao1	490.40	734.71	36.710	0.007
Shannon	3.87	4.09	0.032	0.010
Simpson	0.04	0.03	0.001	0.025

[1)] The treatment group, calves received conventional diet and additives from co-fermented with edible plants and probiotics (30g per head per day).
