# Supplementary material for: The additive from co-fermented edible plants and probiotics improved calves’ growth performance and health by regulating antioxidant and gastrointestinal-microbiota
Source: Anim Biosci. 2025 Nov 14;39(5):250112. doi: 10.5713/ab.250112 (PMC13175069; doi:10.5713/ab.250112)
Supplement: Supplementary file 17 [file ab-250112-Supplement-17.pdf]

Supplement 17. The relative abundance (%) of 10 most predominant phylum and genera in the feces of calves

Items	Control	Treatment[1)]	*P*-value
Phylum			
Firmicutes	65.52±1.675	69.60±2.055	0.010
Actinobacteriota	19.22±2.526	20.73±0.872	0.055
Bacteroidota	9.69±2.502	4.87±0.736	0.025
Cyanobacteria	3.27±1.375	1.58±1.302	0.037
Patescibacteri	1.63±0.491	2.42±1.294	0.337
Proteobacteria	0.50±0.381	0.61±0.185	0.200
Verrucomicrobiota	0.09±0.033	0.04±0.021	0.016
Chloroflexi	0.00±0.001	0.06±0.037	0.004
Spirochaetota	0.02±0.009	0.04±0.033	0.173
Fusobacteriota	0.04±0.023	0.00±0.000	0.007
Others	0.03±0.016	0.05±0.003	0.054
Genus			
norank_f__norank_o__Clostridia_UCG-014	14.82 ± 1.451	20.37±3.052	0.010
Lactobacillus	16.83±4.910	13.43±5.251	0.262
Collinsella	13.64±0.965	13.90±0.889	0.749
Blautia	6.97±3.386	5.70±1.779	0.522
Subdoligranulum	4.39±0.630	7.39±2.617	0.037
Prevotella	7.32±2.577	2.90±0.809	0.025
Bifidobacterium	2.75±0.458	4.13±1.134	0.025
Faecalibacterium	3.16±1.346	2.22±0.730	0.337
norank_f__norank_o__Gastranaerophilales	3.27±1.374	1.58±1.305	0.037
Butyricicoccus	3.25±1.898	1.07±0.609	0.010
Others	23.60±4.330	27.32±2.309	0.078

[1)] The treatment group, calves received conventional diet and additives from co-fermented with edible plants and probiotics (30g per head per day).
