# Supplementary material for: The additive from co-fermented edible plants and probiotics improved calves’ growth performance and health by regulating antioxidant and gastrointestinal-microbiota
Source: Anim Biosci. 2025 Nov 14;39(5):250112. doi: 10.5713/ab.250112 (PMC13175069; doi:10.5713/ab.250112)
Supplement: Supplementary file 18 [file ab-250112-Supplement-18.pdf]

Supplement 18. Significant correlations between fecal bacteria and host phenotypic indicators[1)]

Indicator	Microorganism	Correlation coefficient	*P*-value
ADG	*Butyricicoccus*	-0.77	0.004
	norank_f__norank_o__Gastranaerophilales	-0.67	0.017
Feed efficiency	*Subdoligranulum*	0.74	0.008
	norank_f__norank_o__Clostridia_UCG-014	0.71	0.012
	Butyricicoccus	-0.62	0.035
	norank_f__norank_o__Gastranaerophilales	-0.59	0.049
GSH-pX	*Butyricicoccus*	-0.83	0.001
	norank_f__norank_o__Gastranaerophilales	-0.65	0.026
	Subdoligranulum	0.64	0.028
	norank_f__norank_o__Clostridia_UCG-014	0.64	0.030
	Prevotella	-0.63	0.032
IgA	*Butyricicoccus*	-0.80	0.003
	Prevotella	-0.79	0.004
	norank_f__norank_o__Clostridia_UCG-014	0.73	0.010
	Subdoligranulum	0.65	0.026
IgG	*Bifidobacterium*	0.66	0.022
IL-1β	*Butyricicoccus*	0.59	0.046
MDA	*Butyricicoccus*	0.76	0.004
	norank_f__norank_o__Gastranaerophilales	0.65	0.023
T-AOC	*Bifidobacterium*	0.69	0.017
TNF-α	*norank_f__norank_o__Clostridia_UCG-014*	-0.69	0.017
	norank_f__norank_o__Gastranaerophilales	0.66	0.022
	Subdoligranulum	-0.64	0.028
	Butyricicoccus	0.64	0.030

[1)] Significant correlation = $|R| > 0.5$, $P < 0.05$.
